# *Schistosoma japonicum* extracellular vesicle miRNA cargo regulates host macrophage functions facilitating parasitism

**DOI:** 10.1371/journal.ppat.1007817

**Published:** 2019-06-04

**Authors:** Juntao Liu, Lihui Zhu, Jianbin Wang, Lin Qiu, Yongjun Chen, Richard E. Davis, Guofeng Cheng

**Affiliations:** 1 Shanghai Veterinary Research Institute, Chinese Academy of Agricultural Sciences, Key Laboratory of Animal Parasitology of Ministry of Agriculture, Shanghai, China; 2 Departments of Biochemistry and Molecular Genetics, RNA Bioscience Initiative, University of Colorado School of Medicine, Aurora, Colorado, United States of America; 3 Shanghai Institute of Nutrition and Health, Chinese Academy of Sciences, Shanghai, China; James Cook University, AUSTRALIA

## Abstract

Schistosome infection persists for decades. Parasites are in close contact with host peripheral blood immune cells, yet little is known about the regulatory interactions between parasites and these immune cells. Here, we report that extracellular vesicles (EVs) released from *Schistosoma japonicum* are taken up primarily by macrophages and other host peripheral blood immune cells and their miRNA cargo transferred into recipient cells. Uptake of *S*. *japonicum* EV *miR-125b* and *bantam* miRNAs into host cells increased macrophage proliferation and TNF-α production by regulating the corresponding targets including *Pros1*, *Fam212b*, and *Clmp*. Mice infected with *S*. *japonicum* exhibit an increased population of monocytes and elevated levels of TNF-α. Reduction of host monocytes and TNF-α level in *S*. *japonicum* infected mice led to a significant reduction in worm and egg burden and pathology. Overall, we demonstrate that *S*. *japonicum* EV miRNAs can regulate host macrophages illustrating parasite modulation of the host immune response to facilitate parasite survival. Our findings provide valuable insights into the schistosome-host interaction which may help to develop novel intervention strategies against schistosomiasis.

## Author summary

Schistosomes that cause schistosomiasis infection persist for decades despite a host immune response. Therefore, elucidating the mechanism of schistosome survival will not only contribute to the understanding of host-parasite interaction but also lead to the development of novel strategies against schistosomiasis. Extracellular vesicles (EVs) and their miRNA cargo have been shown to be mediators of intercellular communication involved in the regulation of many biological processes. Here, we demonstrated that EVs released from *Schistosoma japonicum* (SjEVs) are taken up primarily by macrophages and other host peripheral blood immune cells and their miRNA cargo transferred into recipient cells. Uptake of *S*. *japonicum* EV *miR-125b* and *bantam* miRNAs into host cells increased macrophage proliferation and TNF-α production that contributes to parasite survival. Our findings reveal key roles of SjEV miRNAs for facilitating parasitism in schistosomes.

## Introduction

Schistosomes are parasitic flatworms belonging to the genus Schistosoma and are the causative agents of schistosomiasis, a neglected tropical disease [1]. Schistosome infection persists for decades suggesting a complex pathogen-host interaction that likely modulates the host immune response. Schistosomes also utilize host factors for parasite development. For example, host hormones (insulin) [2], growth factors (TGF-β) [3–6] and inflammatory factors [7] have been shown to influence schistosome gene expression, development [8] and metabolism. On the other hand, molecules secreted by the parasites regulate host immunological cell apoptosis [9,10]. Although several studies have evaluated the effect of excreted/secreted products from schistosomes on the host immune response [11–15], the mechanisms that schistosomes use to modulate the functions of host peripheral immune cells remain to be poorly characterized.

Extracellular vesicles (EVs), small membrane-bounded secreted vesicles, are involved in the regulation of many biological processes [16]. Recent studies have described the isolation and characterization of EVs from helminth parasites. For example, proteomic analysis of EVs from *Schistosoma mansoni* indicated that EV proteins are potential vaccine candidates [12,17]; exosomes from *Schistosoma japonicum* induce M1-type immune-activity in macrophages *in vitro* [18], EVs isolated from the trematodes *Echinostoma caproni* and *Fasciola hepatica* are internalized into intestinal host cells [19], and miRNAs are associated with *F*. *hepatica* EVs [20]. However, the regulatory roles of EVs and their miRNA cargo remain poorly characterized. In the nematode *Heligmosomoides polygyrus*, Buck and coworkers identified secreted EVs that contain miRNAs and Y RNA that suppress gene expression in host cells [21], suggesting that helminth EVs as well as their miRNA cargo may play important regulatory roles in parasite-host interactions [22,23].

Adult schistosomes reside in the veins of vertebrate hosts in contact with host peripheral blood immune cells for extended periods. To determine if schistosome EVs regulate the functions of host immune cells, we first isolated SjEVs from adult *S*. *japonicum* and determined their miRNA contents. Using *in vitro* and *in vivo* experiments we then demonstrated that SjEV miRNAs are taken up by host peripheral blood immune cells, particularly by host monocytes. RNA-seq analyses on host cells exposed to SjEVs indicate that SjEV miRNAs may regulate TLR, TNF and other signaling pathways in recipient cells. SjEV miRNA cargo *miR-125b* and *bantam* led to increased monocyte proliferation and TNF-α production by down regulating *Pros1*, *Fam212b* and *Clmp*. Increased monocyte levels induced by SjEVs may be important to the parasites as experiments decreasing monocytes in *S*. *japonicum* infected mice led to markedly reduced worm burden and egg production. Overall, our findings reveal a key role of SjEV miRNA cargo in modulation of host-pathogen interaction that facilitates parasite survival in the definitive host.

## Results

### Characterization of SjEVs and their cargo miRNAs

**Fig 1:**
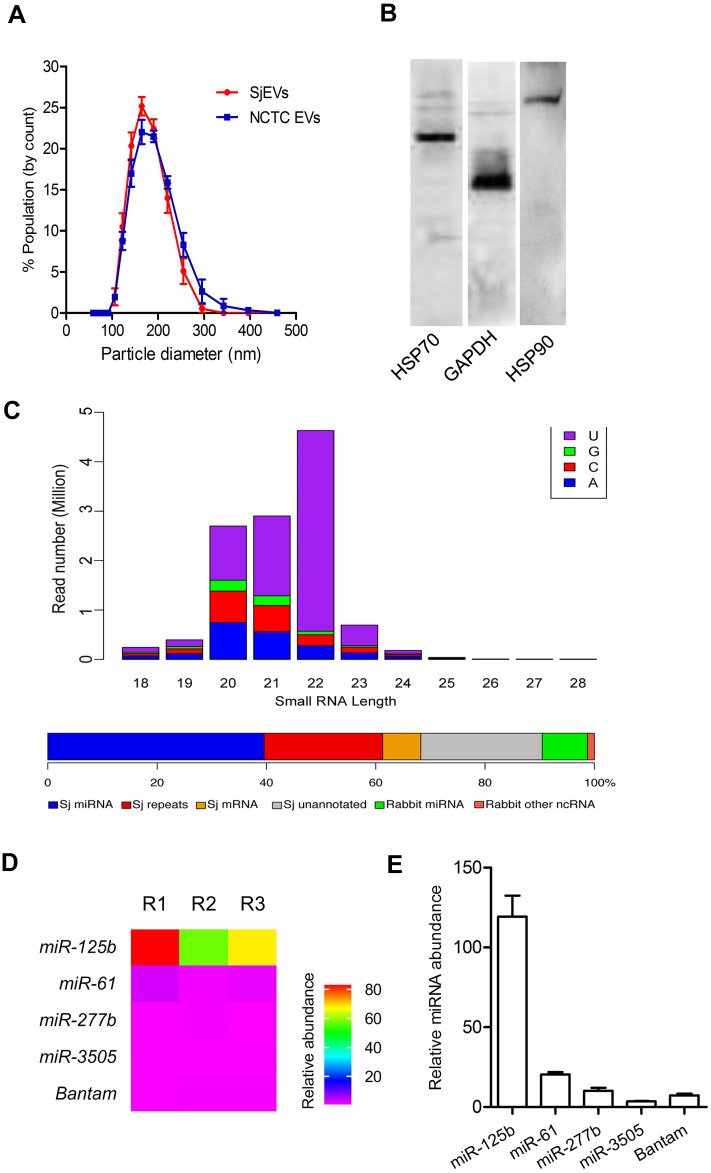
Characterization of SjEVs and their cargo miRNAs. (A) Size distribution of SjEVs and NCTC EVs isolated from culture medium of adult *S*. *japonicum*. The results shown are representative of the data from three independent SjEV/NCTC EV preparations analyzed using a Zetasizer Nano ZS. (B) Immunoblotting analysis of SjEVs contents using antibodies against known mammalian EV markers. (C) Length distribution and first nucleotide (top) of SjEV-associated small RNAs and their classification (bottom) from a representative library. Small RNAs matching to rRNAs and tRNAs were removed from this analysis (see S2 Fig). Sj miRNA: *S*. *japonicum* miRNAs; Sj repeats: small RNAs corresponding to repetitive sequences in the *S*. *japonicum* genome; Sj mRNAs: small RNAs matching *S*. *japonicum* mRNAs; Sj unannotated: small RNAs corresponding to other unannotated sequences in the *S*. *japonicum* genome; Rabbit miRNA: miRNAs that do not match the *S*. *japonicum* genome but correspond to rabbit or other species miRNAs; Rabbit other ncRNAs: small RNAs that do not match the *S*. *japonicum* genome but correspond to the rabbit genome. (D) Heat map of relative abundance of selected SjEV miRNAs. Data illustrate the average of SjEV miRNAs for three biological replications (R1, R2 and R3). (E) RT-qPCR confirmation of the abundance of SjEV miRNAs. Data illustrate representative results and show the mean and standard errors from an experiment carried out in triplicate.

Using a protocol for schistosome EV isolation we previously developed [11,24], we further characterized the isolated SjEVs. qNano analysis indicated that SjEVs ranged in size from 100 nm to 400 nm (Fig 1A). Our previous proteomic studies indicated that the SjEV preparations consist primarily of *S*. *japonicum* proteins [24]. Immunoblotting here indicated that the isolated SjEVs contain several known exosomal markers including GAPDH, HSP70, and HSP90 (Fig 1B). The isolated SjEVs/NCTC EVs were not contaminated with endotoxin (S1 Fig). Next, using high-throughput sequencing we determined the population of small RNAs associated with SjEVs (S1 Table). Bioinformatic analyses indicated that a relatively large percentage of small RNAs in SjEVs were miRNAs (32%) (Fig 1C) including *miR-125b*, *miR-61*, *miR-3505*, and a helminth specific *bantam* miRNA (S1 Table). SjEV *miR-125b* was consistently and highly enriched in SjEVs, accounting for 64.21% of the total miRNA reads in three biological replicates (Fig 1D and S2 Fig). Moreover, using RT-qPCR we verified the relative abundance of several SjEV miRNAs in independently prepared SjEVs (Fig 1E).

### *S*. *japonicum* EVs are primarily taken up by host peripheral blood monocytes and transfer their cargo miRNAs into the recipient cells

**Fig 2:**
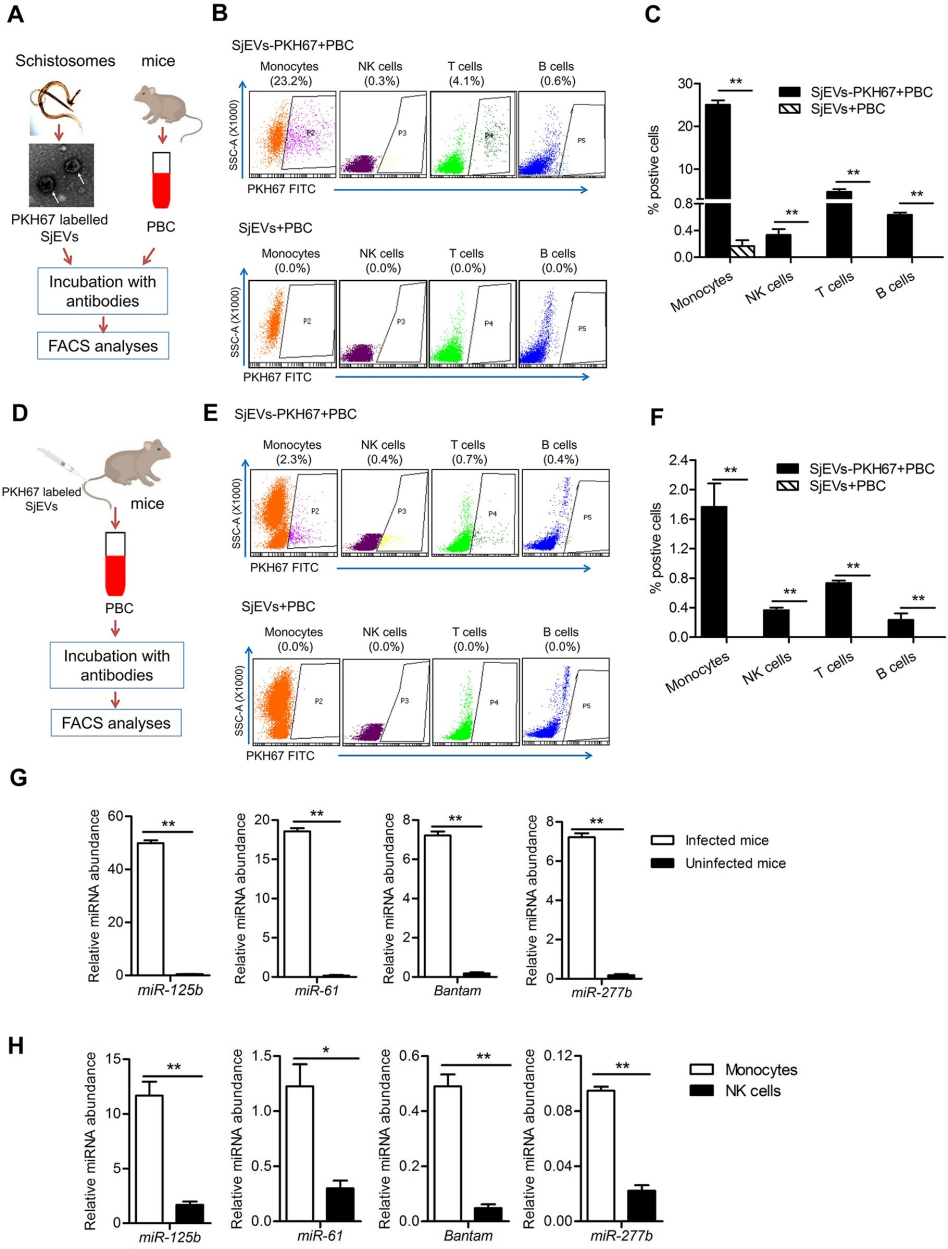
Evaluation of SjEVs and their miRNA cargo uptake by host peripheral blood immune cells. (A) Schematic diagram of experimental approach and analysis of SjEV uptake *in vitro* by murine peripheral blood immune cells. (B) Representative flow cytometry data illustrating *in vitro* SjEV uptake into different populations of murine peripheral blood immune cells. The gating strategies were based on peripheral blood cell incubated with unlabeled SjEVs. The results are representative of three independent experiments. (C) Quantitation of flow cytometry analysis of SjEV uptake in (B). Data illustrate representative results with the mean and standard errors from an experiment carried out in triplicate. (D) Schematic diagram of *in vivo* experiments to determine SjEV uptake in murine peripheral blood immune cells. (E) Representative flow cytometry result analyzing SjEV uptake in different populations of peripheral blood immune cells in mice. The gating strategies were based on peripheral blood cell incubated unlabeled SjEVs. The results are representative of three independent experiments. (F) Quantitation of flow cytometry analysis of SjEV uptake in (E). Data illustrate representative results and show the mean and standard errors from an experiment carried out in triplicate. (G) RT-qPCR of selected SjEV miRNAs in peripheral blood immune cells isolated from *S*. *japonicum* infected mice. (H) RT-qPCR of selected SjEV miRNAs in monocytes and NK cells isolated from *S*. *japonicum* infected mice. Twenty-eight days post-infection, blood samples were collected from mice infected with *S*. *japonicum* and peripheral blood immune cells were sorted by flow cytometry. For panel G and H, data illustrate representative results and show the mean and standard errors derived from 6 mice. * *P* ≤ 0.05 and ** *P* ≤ 0.01.

Adult schistosomes can reside in the host mesenteric veins for several decades in close contact with host peripheral immune cells. To determine whether SjEVs are taken up and regulate host peripheral immune cells (Fig 2A), we incubated *in vitro* PKH67-labeled SjEVs with murine peripheral immune cells. The peripheral immune cells were then sorted using flow cytometry to distinguish CD3e T cells, B220 B cells, CD11b^+^ Ly6C^+^ monocytes, and NK1.1 cells and define the uptake of the labeled EVs into different immune cells. SjEVs were identified predominantly in monocytes, followed by T cells, B cells, and NK cells (Fig 2B and 2C). Experiments using mice injected through the tail vein with PKH67 labeled SjEVs demonstrated that SjEVs were also taken up by peripheral immune cells *in vivo* (Fig 2D). As shown in Fig 2E and 2F and S3 Fig, although uptake appears not as selective *in vivo*, PKH67-labeled SjEVs were highest in monocytes of mice at 4 h and 12 h of post injection consistent with the *in vitro* results. Analysis of peripheral blood immune cells from *S*. *japonicum* infected mice demonstrated that the miRNA cargo of SjEVs, miRNA cargo *miR-125b*, *bantam*, *miR-61*, *miR-277b*, were also taken up and present in peripheral immune cells during a natural infection (Fig 2G). Notably, these *S*. *japonicum* miRNAs are markedly more abundant in monocytes compared to NK cells that take up fewer SjEVs (Fig 2H). Overall, these results indicate that *S*. *japonicum* EVs are taken up primarily by host monocytes with the transfer of their cargo miRNAs into the recipient cells.

### *S*. *japonicum* EV miRNAs are functionally relevant in the recipient cells

**Fig 3:**
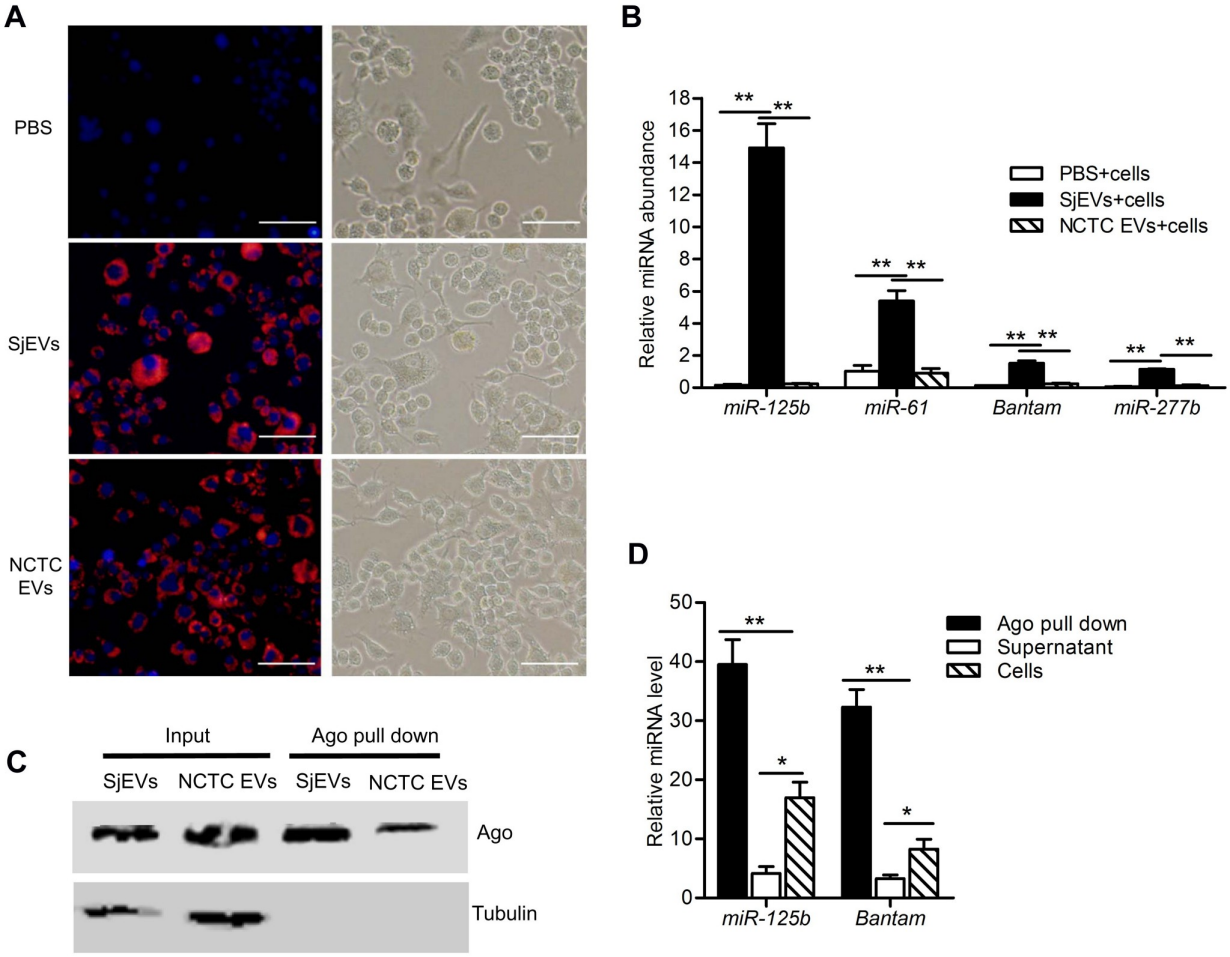
*S*. *japonicum* EV miRNAs are taken up by recipient cells and incorporated into host Argonaute. (A) Uptake of SjEV RNAs by RAW264.7 cells detected using fluorescence microscopy. SjEV RNAs were labeled using the Exo-Glow exosome labeling kit then incubated with RAW264.7 cells. EVs isolated from a murine liver cell line (NCTC clone 1469 cells) were used as a positive control. Red indicates the labeled SjEV RNAs. Nuclei were stained with 4', 6-Diamidino-2-Phenylindole (blue). Bar indicates 50 μm. (B) Detection of SjEV miRNAs in the recipient cells treated with SjEVs by RT-qPCR. (C) Immunoblotting analysis of Argonaute pull down products using Anti-AGO2 and anti-tubulin antibodies. (D) RT-qPCR identification of SjEV *miR-125b* and *bantam* miRNAs are enriched in Argonaute pull down products. For panel B and D, data illustrate representative results and show the mean and standard errors from an experiment carried out in triplicate. * *P* ≤ 0.05 and ** *P* ≤ 0.01.

To initiate experiments to determine if SjEV miRNAs can functionally modulate RNAs within host recipient cells, we first labeled SjEV RNAs using the Exo-Glow exosome labeling kit, incubated the labeled SjEVs with a murine macrophage cell line (RAW264.7 cells), used fluorescence microscopy to examine SjEV uptake into cells, and then determined if SjEV miRNAs were transferred into the recipient cells. As shown in Fig 3A, the labeled SjEVs were taken up by RAW264.7 cells. RT-qPCR analysis indicated that SjEV miRNAs (*miR-125b*, *miR-61*, *bantam*, and *miR-277b*) were readily detected in RAW264.7 cells incubated with SjEVs (Fig 3B), indicating that the SjEV miRNAs were transferred to the recipient cells.

A prerequisite for miRNAs to perform their functions is to be bound by an Argonaute protein, a key component of the RNA-inducing silencing complex (RISC). We used a mammalian Argonaute antibody in a pull-down experiment using RAW264.7 cells treated with SjEVs (Fig 3C) followed by RT-qPCR to determine whether SjEV miRNAs in RAW264.7 cells are loaded into host cell Argonaute. SjEV miRNAs were significantly enriched in Argonaute pull downs (Fig 3D). These results indicate that SjEV miRNAs are transferred to host recipient cells and associate with host cell Argonaute. This suggests that SjEV miRNAs may be incorporated into a functional RISC complex that could regulate host gene expression in the recipient host cells.

### RNA-seq analysis identified differentially expressed mRNAs associated with SjEVs treatment in the recipient cells

**Fig 4:**
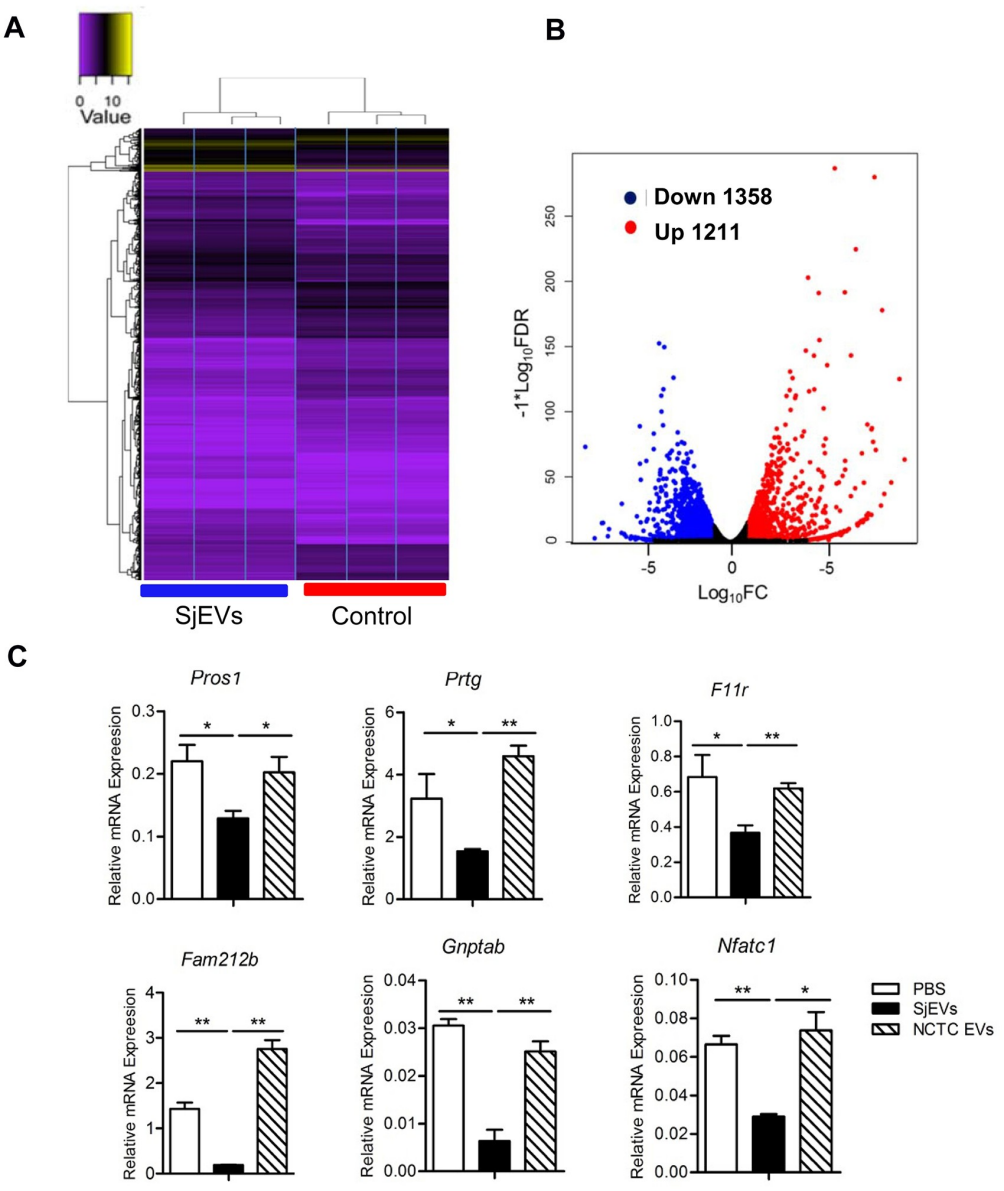
RNA-seq analysis of the differentially expressed mRNAs in SjEVs treated cells. (A) RNA-seq analysis for mRNA expression was performed with total RNA isolated from RAW264.7 cells treated with SjEVs and controls in the three biological replicates. Heat map shows the two-way hierarchical clustering of differentially expressed mRNAs. (B) Volcano plot of mouse genes up- or downregulated upon incubation with SjEVs; red indicates the upregulated mRNAs, while blue indicates the downregulated ones (False Discovery Rate, FDR ≤ 0.05 and logFC (Fold Change) ≥1). (C) Validation of several miRNA predicted target mRNAs in cells treated with SjEVs by RT-qPCR. Data illustrate representative results and show the mean and standard errors from an experiment carried out in triplicate. * *P* ≤ 0.05 and ** *P* ≤ 0.01.

To examine the potential regulatory roles of SjEV taken up by recipient cells, we used RNA-seq to identify changes in steady state levels of mRNAs in cells treated with *S*. *japonicum* EVs. We identified 2,569 affected mRNAs, 1,211 upregulated and 1,358 downregulated, in the three biological replicates of SjEVs treated cells (Fig 4A and 4B and S2 Table). KEGG analysis suggested that altered mRNAs were mainly involved in the TNF and Toll-like receptor (TLR) signaling pathways, cytokine-cytokine receptor interaction, Rap1 signaling pathway and other signaling pathways (S3 Table). To gain further insight into potential regulatory roles of SjEV miRNA cargo in recipient cells, we selected the abundant *miR-125b* (64.21% of the miRNA reads) and *bantam* that is a helminth specific SjEV miRNA for further analysis. We first used TargetScan and miRanda to predict putative targets for these two miRNAs. These programs co-predicted 257 putative mRNA targets for *miR-125b* and 12 putative mRNA targets for *bantam* (S5 Table). We then selected several predicted mRNA targets and used RT-qPCR to corroborate that these mRNA target levels were altered in SjEV treated cells. Consistent with the RNA-seq results, RT-qPCR demonstrated a reduction of these mRNAs in independent experiment of RAW264.7 cells treated SjEVs (Fig 4C). We determined the expressions of several targets in THP-1 monocytes cells treated with SjEVs using RT-qPCR. Similar results were obtained (S4 Fig). In addition, we also examined mRNA levels of several proteins associated with TNF and TLR signaling pathways which we observed to be elevated in the RNA-seq analysis. RT-qPCR analysis confirmed that were increased in cells treated with SjEVs (S5 Fig)

### SjEV *miR-125b* targets *Pros1* to influence the TLR signaling pathway in macrophages

**Fig 5:**
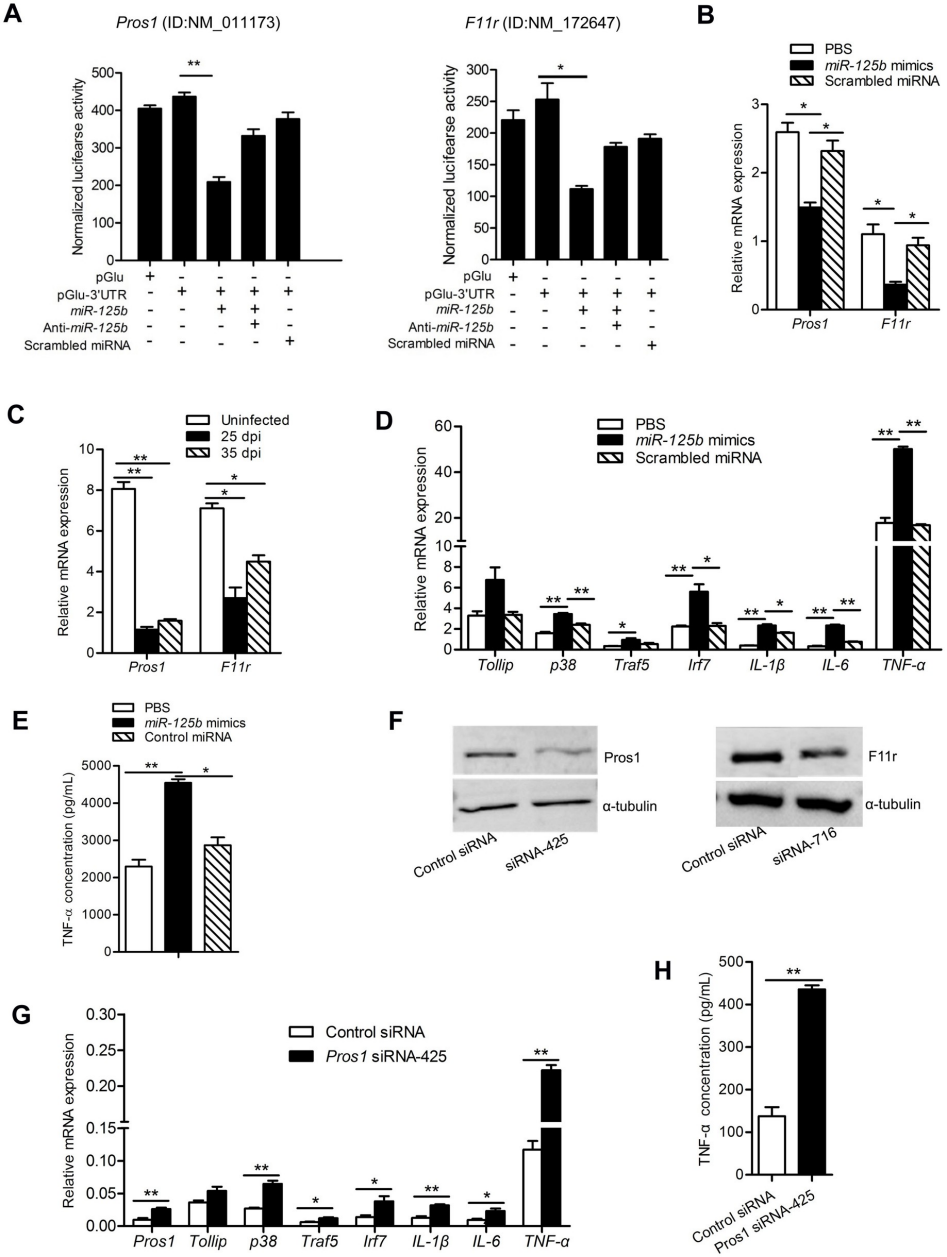
SjEV *miR-125b* targets *Pros1* and influences the TLR signaling pathway in macrophages. (A) Transfection of *miR-125b* mimics into RAW264.7 cells led to a significant reduction of luciferase activity derived from plasmids containing at 3’ UTR with the target of the *miR-125b*. RAW264.7 cells were co-transfected with recombinant or control plasmids (pGlu, empty plasmids; pGlu-3’ UTR, recombinant plasmids with the insertion of 3’ UTR of the target for *miR-125b*) as well as a *miR-125b* mimic, anti-*miR-125b* or a scrambled miRNA control. At 24 h post-transfection, luciferase activity was analyzed using a dual-luciferase reporter assay system with normalization to firefly luciferase levels. (B) RT-qPCR analysis demonstrates reduction of *Pros1* and *F11r* mRNAs in RAW264.7 cells transfected with SjEV *miR-125b* mimics. (C) RT-qPCR analysis of the expression of *Pros1* and *F11r* in peripheral blood monocytes cells isolated from *S*. *japonicum* infected mice (25 dpi: at 25 d post-infection; 35 dpi: at 35 d post-infection). (D) RT-qPCR analysis of mRNA levels for mRNAs related to TLR signaling pathway in RAW264.7 cells transfected with miRNA mimics. (E) ELISA for TNF-α concentration in the culture medium of RAW264.7 cells transfected with SjEV *miR-125b* mimics. (F) Validation of the siRNAs for silencing *Pros1* and *F11r* by immunoblotting analysis. (G) RT-qPCR analysis of the expression of molecules involved in the TLR signaling pathway in *Pros1*-inhibited RAW264.7 cells. (H) ELISA to determine the concentration of TNF-α released from *Pros1*-inhibited cells. Apart from panel F, all experiments show representative results and illustrates the mean and standard errors from an experiment carried out in triplicate. * *P* ≤0.05 and ** *P* ≤ 0.01.

Among the predicted targets of *miR-125b*, protein S1 (Pros1) and F11r are known to inhibit the TLR-triggered inflammatory responses and regulate immune cell functions and cytokine production, respectively. To determine whether *Pros1* and *F11r* mRNAs could be targeted by SjEV *miR-125b*, the mRNA 3’ UTR predicted miRNA-binding sites (predicted using TargetScan and mRanda) were cloned into the 3’ UTR of a luciferase reporter (pGlu-CMV) for evaluation of target repression. The recombinant plasmids as well as the corresponding miRNA mimics and anti-sense were transfected into RAW264.7 cells and luciferase assays carried out. As shown in Fig 5A, transfection of *miR-125b* mimics resulted in a reduction of luciferase activity compared to a scrambled miRNA control, indicating that *miR-125b* can downregulate the expression of an mRNA with the cognate target regions in mammalian cells. Importantly, co-transfection with antisense *miR-125b* mimics in these experiments led to de-repression of luciferase. These results suggest that *miR-125b* can downregulate the expression of the corresponding *Pros1* and *F11r* mRNA target regions in RAW264.7 cells.

To determine whether *miR-125b* can regulate the predicted endogenous cellular targets, we transfected *miR-125b* mimics into cultured RAW264.7 cells and examined the impact on the abundance of their target mRNAs. Transfection of *miR-125b* mimics significantly downregulated *Pros1* and *F11r* mRNA (Fig 5B). Notably, we also observed that both *Pros1* and *F11r* mRNAs were significantly reduced in peripheral blood monocytes isolated from mice infected with *S*. *japonicum* compared to uninfected mice (Fig 5C). Furthermore, transfection of *miR-125b* mimics into RAW264.7 cells also led to an increase in TLR signaling mRNAs including *p38*, *Traf5*, *Irf7*, *IL-1β*, *IL-6* and *TNF-α* (Fig 5D). In addition, transfection of *miR-125b* mimics into RAW247.1 cells resulted in significantly elevated levels of TNF-α in the culture medium (Fig 5E).

To further shown that *Pros1* and *F11r* down-regulation impacts TLR signaling, we designed and optimized siRNAs that effectively silence *Pros1* (siRNA-425) or *F11r* (siRNA-716) (Fig 5F and S6 Fig). Treatment of RAW247.1 cells with the *Pros1* siRNA to inhibit *Pros1* led to increased expression of molecules involved in TLR signaling, including *p38*, *IL-1β*, *IL-6*, *Tlr1*, *Tollip*, *Traf5* and *TNF-α* (Fig 5G). However, *F11r* silencing did not affect TLR signaling (S7 Fig). *Pros1* silencing also led to a significantly increased concentration of TNF-α in the cell culture medium, while no significant effect was observed in *F11r*-silenced cells (Fig 5H and S7 Fig). Overall, these results indicate that SjEV *miR-125b* can downregulate *Pros1* to influence the expression of molecules involved in TLR receptor signaling as well as the expression of TNF-α.

### SjEV *bantam* miRNA targets *Clmp* and *Fam212b* to affect TNF-α production

**Fig 6:**
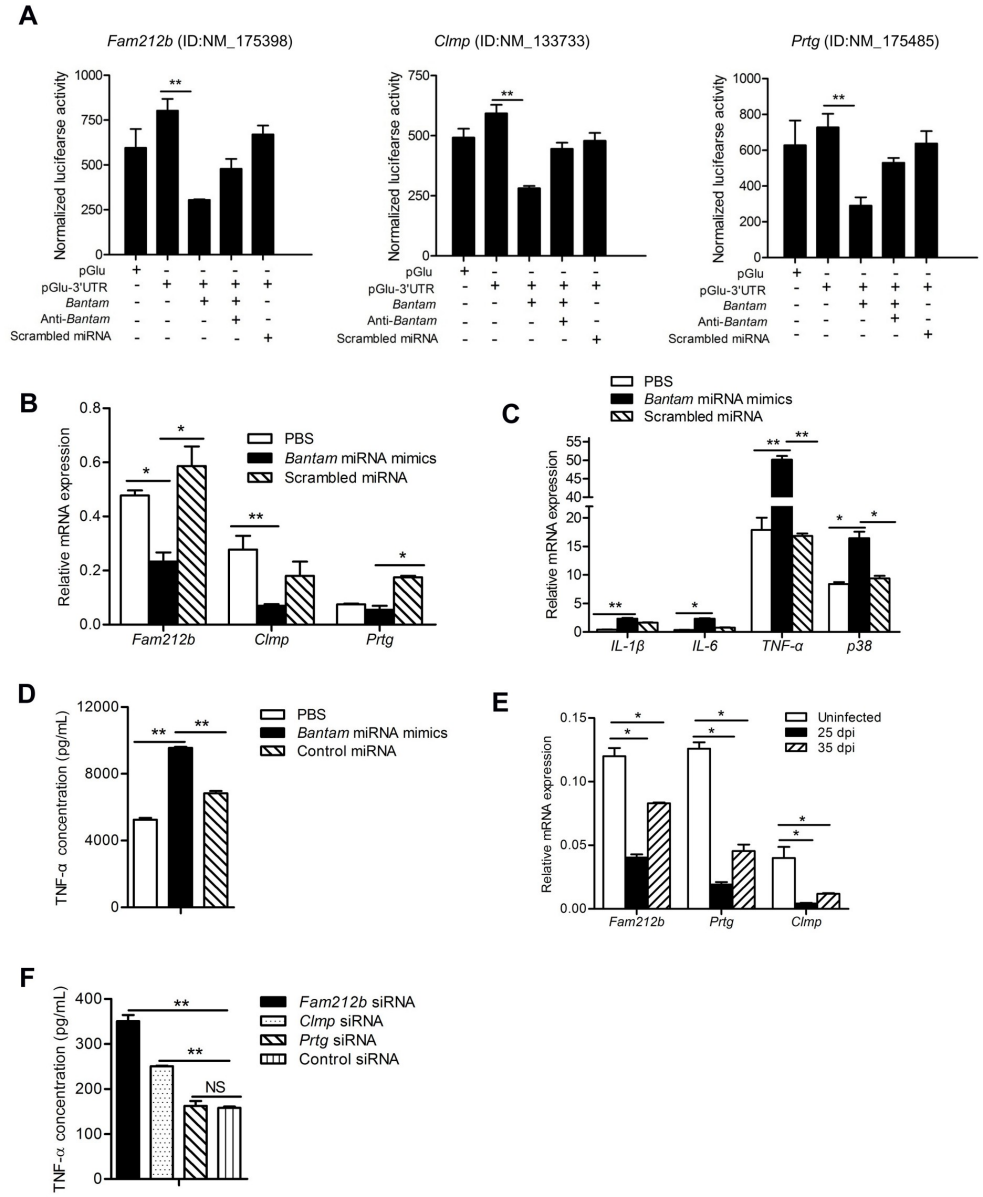
SjEV *bantam* miRNA targets *Clmp* and *Fam212b* to influence TNF-α production. (A) Transfection of *bantam* miRNA mimics in RAW264.7 cells led to a significant reduction of luciferase activity from co-transfected plasmids containing their corresponding target regions. RAW264.7 cells were co-transfected with recombinant or control plasmids (pGlu, empty plasmids; pGlu-3’UTR, recombinant plasmids with 3’ UTR of the target for *bantam*) as well as a *bantam* mimic, anti-*bantam* or a scrambled miRNA control. At 24 h post-transfection, luciferase activity was analyzed using a dual-luciferase reporter assay system with normalization to firefly luciferase levels. (B) *Bantam* miRNA mimic transfection down-regulates its targets in RAW264.7 cells. (C) RT-qPCR analyses of the expression molecules involved in TNF signal pathways in cells transfected with SjEV *bantam* mimics. (D) ELISA assay for TNF-α concentration in the culture medium of RAW264.7 cells transfected with SjEV *bantam* miRNA mimics. (E) RT-qPCR analyses of the expression of *bantam* targets in the peripheral blood monocytes isolated from mice infected with *S*. *japonicum* (25 dpi: at 25 d post infection; 35 dpi: at 35 d post infection). (F) ELISA analysis of the TNF-α concentration in RAW264.7 cells transfected with siRNA duplexes for silencing *Clmp*, *Fam212b*, or *Prtg*. For all panels, data shows representative results and illustrates the mean and standard errors from an experiment carried out in triplicate. * *P* ≤ 0.05 and ** *P* ≤ 0.01.

Among the predicted host cell targets of the SjEV *bantam* miRNA, we selected three including *Fam212b*, *Clmp* and *Prtg* for further analysis, and then cloned the region of each target containing the potential miRNA-binding sites into the 3’ UTR of a luciferase reporter. We found that transfection of *bantam* miRNA mimics resulted in a reduction of luciferase activity of synthetic targets compared to transfection with scrambled miRNA mimics, indicating that *bantam* miRNA can down-regulate the expression of these host target mRNAs (Fig 6A). To determine whether *S*. *japonicum bantam* miRNA can regulate the predicted cellular targets, we transfected *bantam* miRNA mimics into RAW264.7 cells and then examined mRNA levels of the predicted RNA targets in the TNF signal pathway. *Bantam* miRNA mimic transfection led to the downregulation of the predicted targets (Fig 6B) and up-regulated of molecules involved in TNF signaling pathway (Fig 6C). The levels of TNF-α were also significantly increased in the culture medium of cells transfected with *bantam* miRNA mimic (Fig 6D). We also observed that the expression of *Fam212b*, *Clmp*, and *Prtg* were significantly reduced in peripheral blood monocytes isolated from mice infected with *S*. *japonicum* (Fig 6E). Overall, these data suggest that SjEV *bantam* miRNA can target *Fam212b*, *Clmp*, and *Prtg* in macrophages to influence TNF-α production.

To further demonstrate that reduction of *Fam212b*, *Clmp*, and/or *Prtg* mRNAs can affect TNF-α production, we designed and optimized siRNA duplexes for each target (S8 Fig). Introduction of these siRNAs led to reduced levels of *Clmp* and *Fam212b* (but not *Prtg)* and increased expression of TNF-α in macrophages (Fig 6F and S9 Fig). These data support that SjEV *bantam* miRNA downregulation of *Clmp* and *Fam212b* can influence TNF-α expression in macrophages.

### SjEV *miR-125b* and *bantam* influence macrophage proliferation

**Fig 7:**
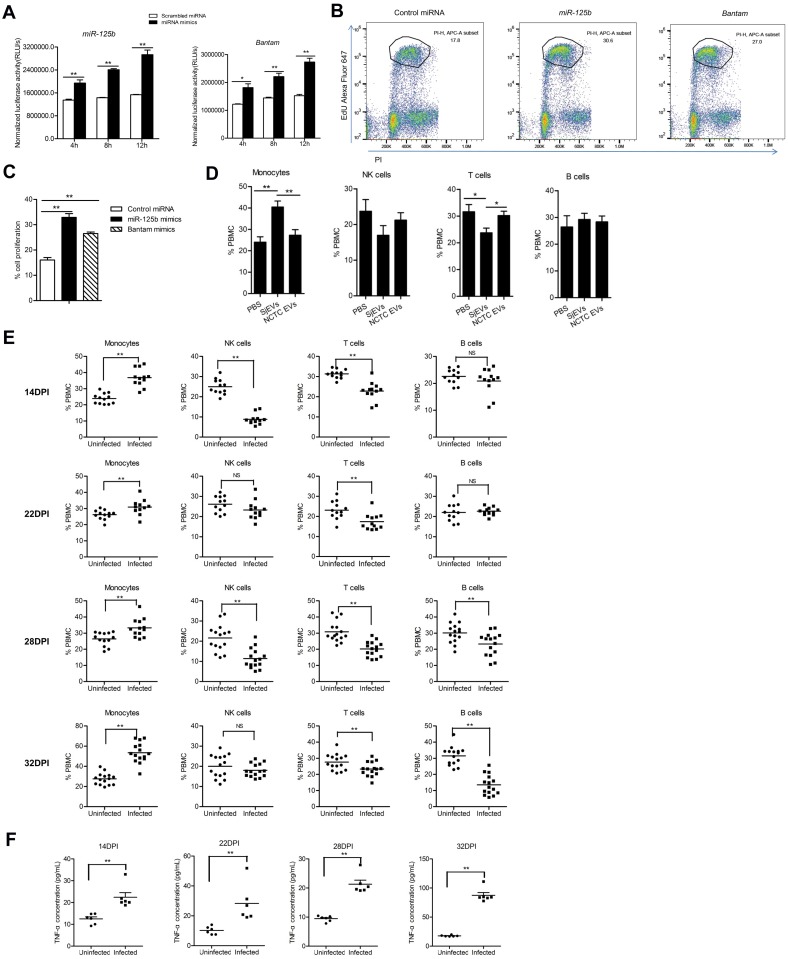
SjEV *miR-125b* and *bantam* influence macrophage proliferation. (A) RAW264.7 cells transfected with SjEV *miR-125b* and *bantam* exhibit increased macrophage proliferation. At the indicated time of post treatment, RAW264.7 cells were collected and assayed. Luciferase activities correspond to levels of cell proliferation. (B) Representative results of flow cytometry analysis of cell proliferation in RAW264.7 cells transfected with SjEV *miR-125b* and *bantam* mimics. (C) Quantitation of flow cytometry analysis of cell proliferation in RAW264.7 cells transfected with SjEV *miR-125b* and *bantam* mimics. The result shows the mean and standard errors from an experiment carried out in triplicate. (D) Injection of SjEVs into mice tail veins leads increases the population of peripheral blood monocytes in mice as determined by flow cytometry analysis. Each experiment shows representative results and illustrates the mean and standard errors derived from 6 mice. (E) The population of peripheral blood monocytes isolated from mice infected with *S*. *japonicum* was increased compared to uninfected mice. Each experiment shows representative results and illustrates the mean and standard errors derived from 10–15 mice. (F) TNF-α levels are increased in sera from mice infected with *S*. *japonicum*. Each experiment shows representative results and illustrates the mean and standard errors derived from 6 mice. For panel A, C, D, E and F, *P ≤ 0.05 and ** *P* ≤ 0.01.

Since SjEV *miR-125b* and *bantam* can increase TNF-α production in macrophages and TNF-α is an autocrine growth regulator for macrophage differentiation, we examined the effect of SjEV miRNA cargo on macrophage proliferation both *in vitro* and *in vivo*. RAW264.7 cells transfected with SjEV *miR-125b* and *bantam* mimics demonstrated significantly increased cell proliferation *in vitro* (Fig 7A). The effects were further confirmed using flow cytometry analysis (Fig 7B and 7C). RAW264.7 cells treated with SjEVs led to increased cell proliferation, whereas the effect was not observed in control treatments such as PBS, NCTC clone 1469 cell EVs and heat-inactivated SjEVs (S10A, S10B and S10C Fig). Further analysis of transcript levels of several M1 and M2 markers such as *iNOS*, *IL-12*, *TNF-α*, *IL-10*, *Arg-1* in RAW264.7 cells treated with SjEVs and protein concentrations of TNF-α, IL-13 and IL-10 in culture media of treated cells indicated that RAW264.7 cells treatment resulted in the mixture of M1- and M2-polarized macrophages (S11 Fig).

To evaluate the effect of SjEVs on macrophage proliferation in mice, we used tail vein injection to deliver SjEVs into monocytes of mice. Flow cytometry analysis indicated that administration of SjEVs significantly increased the population of monocytes, while T-cells decreased, in SjEV treated mice compared to controls (Fig 7D). Next, we determined whether an increased population of peripheral blood monocytes was present in mice infected with *S*. *japonicum*. We found that peripheral blood monocytes were significantly increased (Fig 7E) and the levels of TNF-α were also significantly augmented in serum isolated from *S*. *japonicum* infected mice (Fig 7F) at 14, 22, 28, 32 days of post infection, respectively. Overall, these data demonstrate that SjEVs with their miRNA cargo can increase population of peripheral blood monocytes that may play a critical role in the modulation of host-pathogen interaction.

### Decreased population of host monocytes significantly reduced worm burden and egg deposition in mice

**Fig 8:**
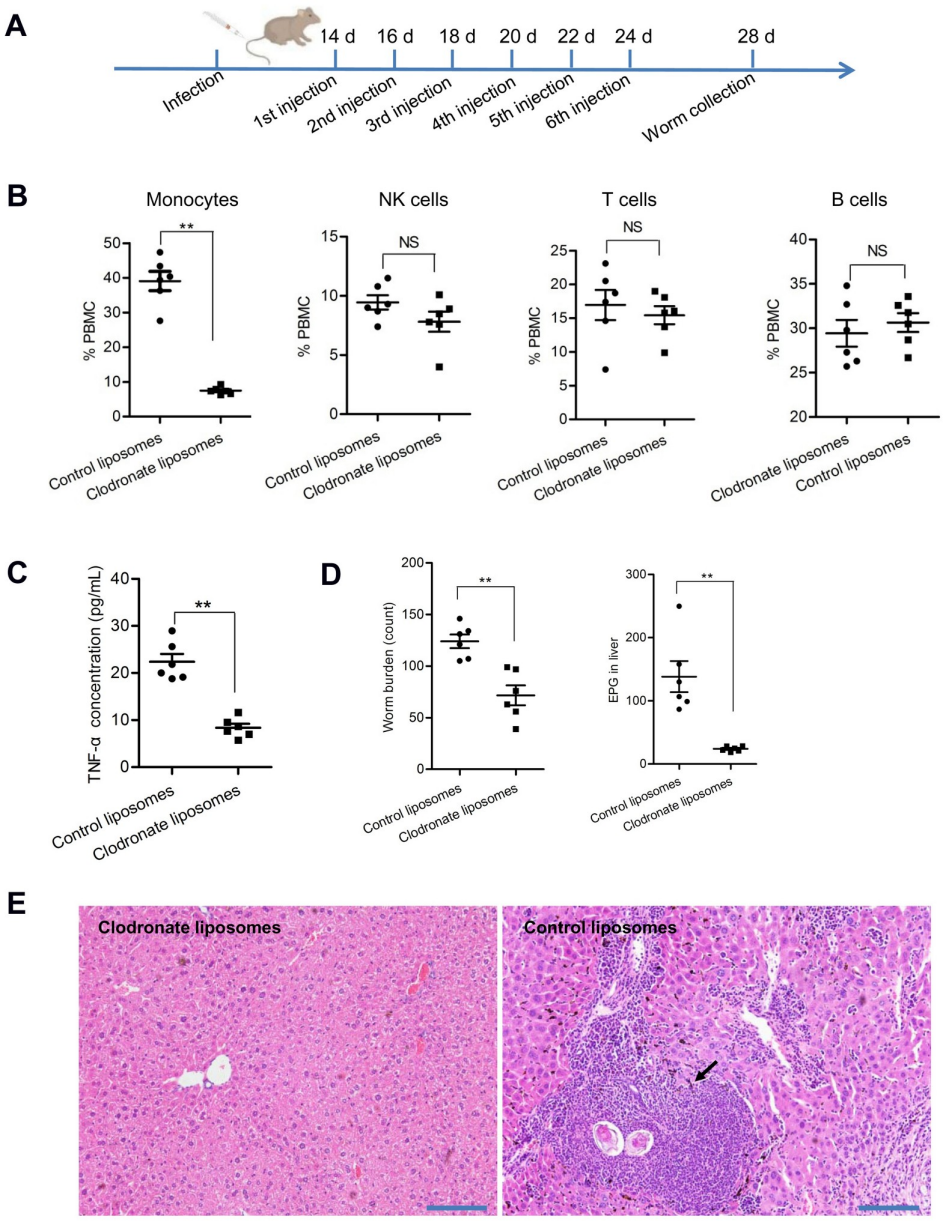
Reducing host monocytes leads to reduced worm burden and egg deposition in *S*. *japonicum* infected mice. (A) Schematic showing liposome injection in mice. Fourteen days post-infection, mice were administered with clodronate liposomes by 6 tail vein injections at two day intervals. (B) Mice treated with clodronate liposomes significantly decreased the population of peripheral blood monocytes (*P* = 0.00000052). At 26 days of post infection, blood was collected from mice administrated with clodronate liposomes and control liposomes and levels of peripheral blood immune cells were analyzed as described in the methods. (C) TNF-α levels were significantly reduced in blood serum isolated from mice administered clodronate liposomes (*P* = 0.00000204). (D) Worm burden and eggs deposited in liver of mice were significantly decreased in mice treated with clodronate liposomes. Each dot represents the number of eggs or parasites collected from each mouse. EPG means eggs per gram of liver. (*P* = 0.0012 for worm burden; *P* = 0.0011 for egg burden). (E) Histological analysis of the liver tissue collected from control and clodronate liposome–treated mice. Arrows indicate the egg-induced granuloma formation. For panel B, C, and D, the data illustrates the mean and standard errors derived from 6 mice. *P ≤ 0.05 and ** *P* ≤ 0.01.

Since an increased population of monocytes and elevated concentrations of TNF-α are associated with mice infected with schistosomes, we evaluated whether host peripheral blood monocyte levels can influence worm burden and egg deposition. Mice were treated with clodronate at day 14 of infection to reduce monocytes (Fig 8A) and at 26 days post-infection, blood samples were collected, and the populations of peripheral blood immune cells were determined by flow cytometry. Monocytes were depleted by approximately 80% in mice administered clodronate liposomes compared to control mice (Fig 8B), and the concentrations of TNF-α were also significantly decreased compared to control mice (Fig 8C). RT-qPCR analysis indicated that the abundance of SjEV *miR-125b* and *bantam* was significantly reduced in monocytes isolated from clodronate liposome–treated mice (S12 Fig).

More importantly, worm burden and egg deposition in the liver was also significantly decreased in mice administered clodronate liposomes (Fig 8D). Egg induced inflammation in the livers was lessened in mice administered clodronate liposomes, compared to the egg-induced granuloma formation in control mice (Fig 8E). Overall, these results suggest that an increased population of peripheral blood monocytes induced by schistosome secreted EVs may play a key role in the parasite survival and egg production.

## Discussion

Adult schistosomes survive in their mammalian host for extended periods in close contact with host peripheral blood immune cells. Understanding the mechanisms through which the parasites manipulate the host immune response for their survival is important to our understanding of the intricate parasite-host interaction and may contribute to the development of novel strategies against schistosomiasis. Here, we demonstrated that SjEVs released from adult *S*. *japonicum* are mainly taken up by host peripheral blood monocytes and that their cargo miRNAs, *miR-125b* and *bantam*, can target host monocyte genes to regulate cell proliferation and TNF-α production. *S*. *japonicum* infected mice exhibit increased levels of monocytes and SjEVs introduced into mice increase host monocytes. SjEVs induction of proliferation of host monocytes and increased TNF-α appears to contribute to schistosome survival. Overall, these findings reveal critical roles of SjEV miRNAs in the modulation of the host immune response that contributes to parasite survival.

In the present study, we isolated extracellular vesicles from *S*. *japonicum* based on an improved protocol as developed in a previous study [24]. Analysis of the size of isolated SjEVs by qNano indicates the particles are 100-400nM in size. The improved protocol may enable isolation of larger EVs such as microvesicles and/or the qNano provides a broader size distribution than our previous EM analysis.

Flow cytometry analysis indicated that SjEVs were predominantly taken up by monocytes (Ly6C^+^ and CD11b^+^) both *in vivo* and *in vitro* studies. Ly6C is an inflammatory marker of monocytes[25]. The inflammatory monocytes typically stay in circulation for short periods (about 1–2 days) and then traffic to the sites of inflammation of tissues and organs. The monocytes can then develop into macrophages that contribute to local and systemic inflammation[26]. Consequently, we used a macrophage cell line (RAW264.7) for our *in vitro* studies to gain insight into the roles of SjEV miRNA cargo on macrophages. We demonstrated that SjEVs are taken up by RAW264.7 cells and alter RNA expression profiles. These changes could be due to protein, small RNAs, lipids, or other material in the SjEVs. Among the *S*. *japonicum* small RNAs in the SjEVs, a large proportion appear to be siRNAs corresponding to repeats followed by miRNAs. A recent study identified repeat associated secondary siRNAs with a nematode Argonaute in the EVs of *Heligmosomodies bakeri* that modulate host genes [27]. In the current study, we focused on SjEV miRNAs including *miR-125b* and *bantam*. We predicted targets of these miRNAs in host cells and demonstrated through transfection of miRNA mimics that a number of host mRNAs can be regulated by the mimics. *SjmiR-125b* can target *Pros1* and *F11r* and *bantam* can target *Clmp*, *Fam212b* and *Prtg*. Previous studies suggested *Pros1* and *F11r* may be involved in the regulation of immune cell functions and cytokine production [28–31]. In addition, *Fam212b*, *Clmp*, and *Prtg* regulate cell adhesion [32], migration [33], or differentiation [34]. Transfection of SjEV *miR-125b* and *bantam* miRNA mimics into RAW264.7 cells led to elevated level of TNF-α and significantly increased expression of molecules involved in TLR and TNF signal pathways. siRNA reduction of *Pros1*, *Fam212b*, and *Clmp* in RAW264.7 cells led to increased levels of TNF-α in the culture medium. Taken together, these results suggest that SjEV *miR-125b* and *bantam* miRNA can regulate TNF-α production by reducing levels of *Pros1*, *Fam212b*, and *Clmp*, and alter macrophage immune cell function.

TNF-α mainly produced by macrophages can increase macrophage proliferation and influence gene expression [35,36]. We show here that mice infected with *S*. *japonicum* have increased levels of monocytes and TNF-α. We further show that RAW264.7 cells transfected with miRNA mimics or treated with SjEVs and SjEV injection into mice leads to proliferation of monocytes and increased TNF-α production. TNF-α has been shown to positively influence parasite development and egg laying [37,38]. We found that reduction of host monocytes using clodronate liposomes introduced into *S*. *japonicum* infected mice led to a significant reduction of worm burden, egg deposition, and TNF-α level (Fig 8C and 8D). Overall, these data suggest that elevated host levels of monocytes and TNF-α may be important for worm development and survival. Consistent with these data, host TNF-α has been shown to induce differential gene expression [39] and protein phosphorylation [7] in schistosomes. In mammalian cells, TNF-α may activate some signaling cascades-caspase cascade with subsequent apoptosis, NF-kB activating cascade and JNK cascade by binding TNF-α receptors [40]. It remains to be determined how increased monocytes and TNF-α influence parasite survival.

Taken together, our results demonstrate that SjEV miRNA cargo can regulate the function of host peripheral monocytes modulating the host immune response to facilitate parasite survival. The findings provide new insights into the role of SjEV miRNAs in schistosome-host interaction and may contribute to develop novel intervention strategies against schistosomiasis.

## Materials and methods

### Ethics statement

All experiments involving mice and rabbits were carried out in strict accordance with the recommendations in the Guide for the Care and Use of Laboratory Animals of the Ministry of Science and Technology of the People’s Republic of China. All efforts were made to minimize animal suffering. All animal procedures were approved by the Animal Management Committee and the Animal Care and Use Committee of the Shanghai Science and Technology Commission of the Shanghai municipal government for the Shanghai Veterinary Research Institute, Chinese Academy of Agricultural Sciences, China (Permit number: SYXK 2016–0010).

### *S*. *japonicum* maintenance, parasite cultures and EV isolation

The life cycle of *S*. *japonicum* (Anhui isolate) was maintained using New Zealand rabbits (Shanghai Ling Chang Biological Technology Co., Ltd, Shanghai, China) and BALB/c mice (Shanghai SLAC Laboratory Animal Co., Ltd, Shanghai, China) and the intermediate snail host *Oncomelania hupensis* (Center of National Institute of Parasitic Disease, Chinese Center for Disease Control and Prevention, Shanghai, China). Mice were challenged with 80–150 normal *S*. *japonicum* cercariae via abdominal skin penetration. New Zealand rabbits were percutaneously infected with approximately 1,500 *S*. *japonicum* cercariae (Anhui isolate, China). Schistosomes were collected at 25–28 days post-infection (dpi) and washed with PBS. The *S*. *japonicum* EVs were isolated as described previously [24]. Briefly, parasites were maintained in preheated RPMI-1640 culture medium for 2 hours at 37°C and 5% CO_2_. The culture media were collected, followed by centrifugation as described previously [24]. A total exosome isolation kit (ThermoFisher Scientific, Carlsbad, CA, USA) was used for EV isolation according to the manufacturer’s instructions with minor modifications as described previously [24]. The size of isolated EVs was determined using Zetasizer Nano (Malvern, United Kingdom). The endotoxin activity of isolated EVs was determined using ToxinSensor Chromogenic LAL Endotoxin Assay Kit according to the manufacturer’s instructions (GenScript, Nanjiang, China).

### Small RNA library preparation and bioinformatic analysis

Total RNA was extracted from *S*. *japonicum* EVs using TRIzol LS reagent (ThermoFisher Scientific), and RNA quality was analyzed using an Agilent 2100 system (Agilent Technologies, Santa Clara, CA, USA). RNA was size-selected using 15% denaturing PAGE, and libraries were prepared from the 18–30 nt fraction using an TruSeq Small RNA Library Preparation Kit (Illumina, San Diego, CA, USA). The small RNA libraries were subjected to sequencing using Illumina 50bp single end sequencing performed on an Illumina HiSeq 2000 machine at the Beijing Genomics Institute (Shenzhen, China). The raw sequencing data were deposited with the NCBI SRA accession: PRJNA508449.

Reads were mapped to the draft *S*. *japonicum* genome sequences (sjr2_scaffold.fasta, downloaded from ftp://lifecenter.sgst.cn:2121/nucleotide/corenucleotide) using the Short Oligonucleotide Alignment Program (http://soap.genomics.org.cn). Reads were also mapped to the rabbit genome (http://asia.ensembl.org/Oryctolagus_cuniculus/, version 81) to identify host sequences associated with the *S*. *japonicum* EV library.

Small RNAs from each library were sequentially mapped to the following databases for their classification: (1) Sj rRNAs and tRNAs; (2) Sj miRNAs; (3) Sj repeats; (4) Sj mRNAs; (5) Sj genome; (6) Rabbit rRNAs and tRNAs; (7) Rabbit miRNAs and other miRNAs from miRBase (v21); and (8) Rabbit genome. The results were derived using bowtie [40] mapping allowing one mismatch. We also compared mapping results that allows no mismatch as well as 2 or 3 mismatches. The number of the reads that can be mapped increase as the criteria for matching becomes less stringent, but the overall conclusion is the same. This is consistent with sequence polymorphisms derived from different individuals as compared to the reference genome.

### Immunoblotting

The isolated SjEVs were lysed using lysis buffer, separated by 10% SDS-PAGE, and immunoblotting was performed as described previously [25] using primary antibodies against HSP70 (Catalog number: D154145-0025), GAPDH (Catalog number: D110016-0100) (Sangon Biotech, Shanghai, China) and HSP90 that produced by immunizing purified recombinant SjHSP90 protein (1:2000 dilutions).

### Uptake of *S*. *japonicum* EV RNAs by macrophage

Murine macrophage (RAW264.7) cells were obtained from the American Type Culture Collection (ATCC) and grown using DMEM (ThermoFisher Scientific) medium supplemented with 10% fetal bovine serum (FBS, ThermoFisher Scientific). Murine liver cells (NCTC clone 1469 cells) were obtained from the ATCC and grown using DMEM (ThermoFisher Scientific) medium supplemented with 10% horse serum (ThermoFisher Scientific). EVs were isolated from the supernatant of NCTC clone 1469 cells. Briefly, cell supernatants were harvested, centrifuged at 2,000 × *g* for 30 min to eliminate cells, and then centrifuged at 12,000 × *g* for 20 min to eliminate cellular debris and larger vesicles. EVs were isolated as described above. The protein concentration of EVs was determined using a BCA protein assay kit (Sangon Biotech, Shanghai, China).

To verify the internalization of *S*. *japonicum* EVs by RAW264.7 cells, cells were seeded in 12-well plates (approximate 2×10^5^ cells per well) and cultured with advanced DMEM serum-free media (ThermoFisher Scientific) for 4 h. The EVs from *S*. *japonicum* (5 μg) or NCTC clone 1469 cells (5 μg) were labeled using a Exo-Glow exosome labeling kit (System Biosciences, Palo Alto, CA, USA) according to the instructions as described by Peterson *et al*. (2015)[41], in which the RNAs associated with EVs are labeled red. The labeled SjEVs were incubated with RAW264.7 cells for 4 hours. Then, cells were fixed by 4% paraformaldehyde, washed with PBS, and stained with 4',6-Diamidino-2-Phenylindole (1μg/mL) (ThermoFisher Scientific), the cells examined by fluorescent microscopy and the images captured (Olympus, Tokyo, Japan).

### Flow cytometry

Antibodies directed against the following markers were used to stain peripheral blood immune cells for flow cytometry analysis: Ly-6C-APC (Clone: HK1.4, Catalog number: 17-5932-80), CD11b-APC-eFluor780 (Clone:M1/70, Catalog number: 47-0112-80), CD45R(B220)-eFluor450 (Clone: RA3-6B2, Catalog number: 48-0452-80), NK1.1 PE-eFluor 610 (Clone: PK136, Catalog number: 61-5941-80), and CD3e PE (Clone: 145-2C11, Catalog number: 12-0031-81). All antibodies were obtained from eBioscience (San Diego, CA, USA). To determine SjEV uptake into peripheral blood immune cells, 200 μL of blood was collected from mice with anticoagulant sodium citrate solution, red cells lysed using RBC lysis buffer (Biolegend, San Diego, USA), the remaining cells incubated with 2.5 μg PKH67 labeled SjEV for 4 h, stained with antibodies (1:200 dilution) at 4 °C for 30 min., and then the cells were washed with 1% BSA wash solution. The cells were suspended in 200 μL of wash solution and then sorted and analyzed using a BD FACSAria II system (BD Biosciences, Mountain View, CA, USA). Each measurement contained 1×10^5^ cells. Data were analyzed using FACSDiva software (BD Biosciences).

To determine the distribution of SjEVs in mice peripheral blood immune cells, mice were administered PKH67-labeled SjEVs (5 μg/mice) by tail vein, and four hours and twelve hours post-injection, blood was collected and peripheral blood immune cells were isolated, analyzed, and sorted by flow cytometry as described above. The percentage of cells positive for PKH67-labeled SjEVs was determined for each cell population.

To characterize peripheral blood immune cells in mice, 200 μL of blood was collected from control mice and mice infected with *S*. *japonicum* and then incubated with the antibodies described above and CD45 monoclonal antibody-FITC/eFluor506 (Clone: 30-F11, Catalog number: 11-0451-81) (eBioscience). The cells were treated with red cell lysis buffer (Biolegend) as described above. For some experiments, FACS-sorted monocytes, NK cells, T cells and B cells were collected and total RNAs were isolated and RT-qPCR performed as described below.

### RNA isolation and RT-qPCR

Total RNA was extracted from samples using TRIzol reagent (ThermoFisher Scientific) according to the manufacturer’s protocol. RNA was quantified using a Nanodrop ND-1000 spectrophotometer. For miRNA analysis, the miScript II RT Kit (Qiagen, Hilden, Germany) was used to reverse-transcribe RNA to cDNA. Real-time PCR was performed using a miScript SYBR Green PCR Kit (Qiagen) in an Eppendorf Realplex2 Detection System Mastercycler ep realplex (Eppendorf, Hamburg, Germany). The PCRs were performed at 95 °C for 15 min, followed by 45 cycles of 94°C for 15 s, 55°C for 30 s, and 70°C for 30 s. The miScript primers for *miR-125b*, *bantam*, *miR-61*, *miR-3505* and *miR-277b* are the property of Qiagen. For determining the abundance of miRNAs in SjEVs, spike-in control of *C*. *elegans miR-39* miRNA mimic provided from miRNeasy Serum/Plasma Spike-In Control (Qiagen) was used as the control. For determining the abundance of SjEV miRNAs in the RAW264.7 cells, *Glyceraldehyde 3-phosphate dehydrogenase* (*GAPDH*) was used as the internal control (forward primer: CAT GGC CTT CCG TGT TCC TA; reverse primer: CCT GCT TCA CCA CCT TCT TGA T). The 2^−ΔCt^ method was used to calculate relative miRNA abundance [42].

For mRNA expression analysis in RAW264.7 cells or peripheral blood monocytes cells, cDNAs were transcribed from total RNA with random primers combined with oligo dT primer using a PrimeScript RT reagent Kit (TaKaRa, China), and RT-qPCR analysis was performed using the SYBR Premix ExTaq kit (TaKaRa) according to the manufacturer’s instructions. Primer sequences are shown in S4 Table. The murine *GAPDH* gene was used as the internal control. The 2^−ΔCt^ method was used to calculate relative mRNA abundance as described above.

For mRNA expression analysis in THP-1 cells, THP-1 cells were kindly provided by Stem Cell Bank of Chinese Academy of Sciences and were cultured with RPMI Medium 1640 containing 10% of heat inactivated fetal bovine serum (ThermoFisher Scientific) and supplemented with 10 mM Hepes (ThermoFisher Scientific) and 50 pM β-mercaptoethanol (ThermoFisher Scientific). The SjEVs and NCTC EVs were incubated with THP-1 cells for 4 h. The total RNA isolation, cDNA preparation and qPCR as described above. The *β-Actin* gene was used as the internal control. Primers sequences are shown in S4 Table.

### Argonaute Immunoprecipitation

RAW264.7 cells were seeded in 6-well plates (2×10^5^ cells per well) and cultured overnight. The following day, cells were treated with SjEVs (5 μg/well) or NCTC clone 1469 cell EVs (5 μg/well). Cell lysates were prepared in 500 μL of lysis buffer containing 25 mM Tris (pH 7.4), 150 mM KCl, 0.5% NP-40, 2 mM ethylenediaminetetraacetic acid (EDTA), 1 M NaF, 0.5 M dithiothreitol, and protease inhibitors and then centrifuged at 10,000×g for 10 min at 4 °C. The pull-down assay was performed using a Dynabeads Protein G Immunoprecipitation Kit (ThermoFisher Scientific) according to the manufacturer’s instructions with Anti-AGO2 antibody (Abcam, Catalog number: ab186733). In brief, approximately 10 μL of Anti-AGO2 antibody was coupled with 1.5 mg of Dynabeads. Then, 200 μL of the protein lysate (100 μg of protein) was incubated with the antibody-treated beads for 20 min at room temperature, and the Dynabeads were washed three times in 200 μL of the wash buffer provided with the kit. Finally, target antigens were eluted with elution buffer, the elutes separated by SDS-PAGE, and then analyzed by immunoblotting using Anti-AGO2 antibody and tubulin antibody (Catalog number: T6074, Sigma). RNA was also isolated from the immunoprecipitates and qPCR was used to examine SjEV *miR-125b* and *bantam* levels.

### RNA-seq and bioinformatic analyses

RAW264.7 cells were grown in DMEM as described above and seeded into 24-well plates at approximately 20,000 cells per well. The following day, cells were incubated with *S*. *japonicum* EVs (5 μg total EV protein per well) for 4 h and then washed twice with PBS. Total RNA was extracted using TRIzol reagent (ThermoFisher Scientific) according to the manufacturer’s protocol. The quality of RNA was determined using an Agilent 2100 system (Agilent Technologies). Library preparation and sequencing were carried out at Shanghai Personal Biotechnology Co., Ltd. (Shanghai, China). Polyadenylated mRNAs were isolated from the total RNA using beads with oligo (dT) (ThermoFisher Scientific), the mRNA fragmented, and cDNA prepared using random hexamer primers. A cDNA library was then prepared using a SureSelect Strand Specific RNA Library kit (Agilent Technologies) according to the manufacturer’s specifications. Paired-end reads with an average length of 101 bp were generated by sequencing cDNA libraries on an Illumina HiSeq 2000 sequencer (Illumina).

Raw data were filtered to remove low-quality reads and the reads mapped to reference sequences (http://asia.ensembl.org/Mus_musculus/info/index) using the Short Oligonucleotide Alignment Program (http://soap.genomics.org.cn). Raw data files have been deposited at the NCBI under project number PRJNA471019.

### Target prediction and bioinformatics analyses

In *silico* prediction of potential miRNA target genes was carried out by miRanda (http://www.microna.org/microrna/getDownloads.do) and TargetScan (http://www.targetscan.org/mmu_71/). Target genes predicted by miRanda and TargetScan that were also observed to be downregulated in *S*. *japonicum* EVs treated RAW264.7 cells were selected for further analysis.

### Plasmid construction and purification

Target 3’ UTR mRNA fragments corresponding to SjEV *miR-125b* targets derived from miRanda and TargetScan were PCR-amplified from *S*. *japonicum* cDNA, and the PCR products were cloned into the pCMV-Glu vector 3’ UTR (Targeting Systems, El Cajon, CA, USA) using standard molecular cloning methods. The primer pairs used for PCR amplification and the restriction enzyme sites are listed in S6 Table. The recombinant plasmids were confirmed by sequence analysis.

### Cell transfection

Recombinant and control plasmids were transfected at 80 ng per well (approximately 2×10^4^ cells) into cultured HEK293/RAW264.7 cells using Lipofectamine 2000 (ThermoFisher Scientific) along with pGL3 (40 ng) for normalization. At 24 h post-transfection, the cells were further transfected with a miRNA mimic, anti-miRNA, or control scrambled miRNA (40 nM) (S7 Table) at the indicated concentrations using Lipofectamine 2000. The miRNA mimics, anti-sense miRNAs or scrambled miRNAs used were modified by 2`-O-methyl or 2`-O-methyl and phosphorothioate. The transfected cells were incubated for the indicated time prior to collection for dual luciferase assay as described below. Transfections were carried out in triplicate three times using two independent plasmid preparations.

### Luciferase assays

At 24–48 h post-transfection, luciferase assays were carried out using the Dual Glo Luciferase assay system (Promega, Madison, WI, USA) according to the manufacturer’s instructions, and luminescence was measured by a luminometer (Berthold, Germany). The relative reporter activity for transfected cells was obtained by normalization to co-transfected firefly luciferase activity (pGl3) or protein concentration using the Pierce BCA protein assay kit combined with the Compat-Able protein assay preparation reagent set (ThermoFisher Scientific).

### Cell proliferation and polarization

RAW264.7 cells were incubated with SjEVs, PBS, NCTC clone 1469 cell EVs, or transfected with miRNA mimics or control miRNA (S7 Table). At the indicated times, cell proliferation was analyzed using a Cell Titer-Lumi Luminescent cell viability assay kit (Beyotime Biotechnology, Jiangsu, China) according to the manufacturer’s protocol. The luciferase activity was normalized to protein concentration using the Pierce BCA protein assay kit combined with the Compat-Able protein assay preparation reagent. For flow cytometry analysis, a Click-iT EdU Alexa Fluor 647 Flow Cytometry Assay Kit (ThermoFisher Scientific) was used to determine cell proliferation according to the manufacturer’s protocol. Briefly, RAW264.7 cells were seeded in 24-well plates (approximate 2×10^4^ cells per well) and cultured overnight. The following day, cells were treated with SjEVs, NCTC clone 1469 cell EVs, or PBS or transfected with *miR-125b* and *bantam* miRNA mimics for 5 hours. EdU was then added to the culture medium at a final concentration of 10 μM and incubated for 2 h. The cells were then washed with 1% BSA, fixed using Click-iT Fixative and incubated for 15 min at room temperature. The fixed cells were washed twice with 1% BSA and resuspended in 100 μL of 1X Click-iT saponin-based permeabilization and wash reagent. Click-iTreaction cocktail (0.5 ml) was added to each tube, mixed well and incubated for 30 min at room temperature. The cells were washed with 3 mL of 1X Click-iT saponin-based permeabilization and wash reagent and then resuspended in 500 μL of 1X Click-iT saponin-based permeabilization and wash reagent for flow cytometry analysis.

To determine macrophage polarization upon SjEV treatment, RAW264.7 cells were treated with 1 μg/ml LPS (Sigma-Aldrich, St. Louis, MO, USA), 10 ng/ml IL-4 (R&D Systems, Minneapolis, MN, USA), SjEVs, NCTC EVs, PBS (blank control). At 12 h of post treatment, total RNAs were isolated from treated cells and prepared cDNA was used for qPCR analysis as described above. The culture media of cells treated with SjEVs, NCTC EVs and PBS were analyzed for TNF-α, IL-10, and IL-13 concentrations as described below.

### RT-qPCR and immunoblotting analysis of target gene silencing

RAW264.7 cells were seeded in 24-well plates (approximate 2×10^4^ cells per well) and cultured as described above. On the following day, siRNAs targeting *Pros1*, *F11r*, *Prtg*, *Fam212b*, *Clmp* and control siRNA were transfected into cultured cells (S8 Table). At 24–48 hours post-transfection, the cells were collected for RT-qPCR or protein analysis. The qPCR primers are listed in S4 Table. Commercially available antibodies for Pros1 and F11r were used for immunoblotting as described above, with the primary antibodies against Pros1 (Catalog number: D121412-0025), F11r (Catalog number: D154077-0025) (Sangon Biotech, Shanghai, China), and tubulin (Catalog number: T6074, Sigma) antibodies at a 1:800 dilution.

### ELISA for quantitative detection of TNF-α and other cytokines

To quantify TNF-α concentration, cell culture media from different treatments or murine sera isolated from controls and schistosome infected mice at different times of post-infection were analyzed by the TNF-α Mouse ELISA Kit (ThermoFisher Scientific) according to the manufacturer’s protocol. Briefly, 50-μL samples (medium or sera) and diluted standards were added into wells, and then, 50 μL of biotin conjugate was added into each well. The plates were covered with adhesive film and incubated at room temperature for 2 hours on a microplate shaker. Then, the wells were washed six times with wash buffer. Next, 100 μL of diluted streptavidin-HRP was added to wells and then incubated at room temperature for 1 hour. After six washes, 100 μL of TMB substrate solution was added into each well, incubated at room temperature for 30 min, and then stop solution was added. The optical density in each well was measured by a TECAN microplate reader (TECAN, Switzerland). In addition, aliquots from cell culture supernatants were used to analyze TNF-α, IL-10, and IL-13 using ELISA kits, according to the manufacturer’s instructions (eBioscience, USA). Cytokine concentrations were calculated by referring to standard curves.

### Schistosome infection of mice

Four- to six-week-old male BALB/c mice (mean weight 25 ± 2 g) were purchased from the Shanghai SLAC Laboratory Animal Co., Ltd. Twelve mice were randomly divided into two groups. Each mouse was challenged with 150 ± 5 normal *S*. *japonicum* cercariae via abdominal skin penetration. At 14 days post-infection, mice in each group were injected with 200 μL of clodronate liposomes (from Vrije Universiteit Amsterdam) or control liposomes (from Vrije Universiteit Amsterdam) diluted in 800 μL of PBS via the tail vein. Five additional injections were performed at 16, 18, 20, 22 and 24 days post-infection. At 26 days post-infection, blood samples were collected from each mouse, and peripheral blood immune cells were sorted by flow cytometry as described above. At 28 days post-infection, the parasites were perfused, and worm burden and egg production in the liver were counted as described elsewhere [43]. The abundance of SjEV *miR-125b* and *bantam* in monocytes were determined by RT-qPCR as described above. The concentration of TNF-α in the serum of mice was measured by ELISA as described above.

For SjEVs tail vein injection experiments, mice were administered with either SjEVs (5 μg/mouse) in 100 μl PBS, NCTC clone 1469 cell EVs (5 μg/mouse) in 100 μl PBS, or 100 μl PBS. At 4–24 hours of post-injection, blood was collected, and peripheral blood immune cells were sorted by flow cytometry as described above.

### Histochemical section analysis

Mouse livers collected from the monocyte depletion experiments were cut into approximately 1.0 cm×1.0 cm×0.3 cm pieces, washed in PBS, and fixed in 10% formalin. The formalin-fixed samples were dehydrated in ethanol, cleared with xylene and embedded in paraffin wax. Sections (6 μm thick) were prepared and stained with hematoxylin and eosin.

### Statistical analysis

Results were analyzed using SPSS software (version 17). Comparisons between groups were made using Student’s t tests / one way ANOVA. Differences were considered significant when *P* ≤ 0.05.

## Supporting information

S1 Fig Analysis of endotoxin activity associated with isolated SjEVs and NCTC EVs (Data represents the results obtained from the five biological replicates of isolated SjEVs and NCTC EVs). (TIF)

S2 Fig Length distribution, first nucleotide, and classification of SjEV-associated small RNAs (data represents three biological replicates). (TIF)

S3 Fig Evaluation of SjEVs uptake by host peripheral blood immune cells in mice *in vivo* at 4 h of post injection. (A) Representative flow cytometry result analyzing SjEV uptake in different populations of peripheral blood immune cells in mice. The gating strategies were based on peripheral blood cell incubated unlabeled SjEVs. The results are representative of six independent experiments. (B) Quantitation of flow cytometry analysis of SjEV uptake in (A). Data illustrate representative results and show the mean and standard errors from six mice. * *P* ≤ 0.05 and ** *P* ≤ 0.01.
(TIF)

S4 Fig Validation of several miRNA predicted target mRNAs in THP-1 cells treated with SjEVs by RT-qPCR. Data illustrate representative results and show the mean and standard errors from an experiment carried out in triplicate. * *P* ≤ 0.05 and ** *P* ≤ 0.01.
(TIF)

S5 Fig Validation of some differentially expressed mRNAs related to TLR and TNF signaling pathways in the cells treated with SjEVs by RT-qPCR. Data illustrate representative results and show the mean and standard errors from an experiment carried out in triplicate. * *P* ≤ 0.05 and ** *P* ≤ 0.01.
(TIF)

S6 Fig Screen of the best siRNA duplex for silencing *Pros1* and *F11r*. Each experiment shows representative results and illustrates the mean and standard errors from an experiment carried out in triplicate. * *P* ≤ 0.05 and ** *P* ≤ 0.01.
(TIF)

S7 Fig Effect of *F11r* inhibition on the expression of molecules involved in TLR signal pathways and TNF-α concentration. (A) RT-qPCR analysis of the expression of molecules involved in the TLR signaling pathway in RAW264.7 cells transfected with *F11r* siRNA-716. Each experiment shows representative results and illustrates the mean and standard errors from an experiment carried out in triplicate. * *P* ≤0.05. (B) ELISA for TNF-α concentration in the culture medium of RAW264.7 cells transfected with *F11r* siRNA-716. Each experiment shows representative results and illustrates the mean and standard errors from an experiment carried out in triplicate.
(TIF)

S8 Fig Screen of the best siRNA duplex for silencing *Fam211b*, *Clmp* and *Prtg*. Each experiment shows representative results and illustrates the mean and standard errors from an experiment carried out in triplicate. * *P* ≤ 0.05 and ** *P* ≤ 0.01.
(TIF)

S9 Fig RT-qPCR analyses of the transcript levels of TNF-α in RAW264.7 cells transfected with siRNA duplexes for silencing *Clmp*, *Fam212b*, or *Prtg*. Data shows representative results and illustrates the mean and standard errors from an experiment carried out in triplicate. * *P* ≤ 0.05 and ** *P* ≤ 0.01.
(TIF)

S10 Fig RAW264.7 cells treated with SjEVs increased cell proliferation. (A) SjEVs treatment of RAW264.7 cells increases their proliferation. At the indicated time of post treatment of SjEVs, RAW264.7 cells were collected and assayed using a cell Titer-Lumi luminescent cell viability kit. The luciferase activities indicated cell proliferation was increased as compared to that treated with heated inactivated SjEVs. Each experiment shows representative results and illustrates the mean and standard errors derived from triplicate experiments from an experiment carried out in triplicate. (B) and (C) SjEV treatment of RAW264.7 cells increases the population of cells in S phase. Each experiment shows representative results and illustrates the mean and standard errors from an experiment carried out in triplicate.
(TIF)

S11 Fig Analysis of the expressions of several M1/M2 markers in RAW264.7 cells treated with SjEVs. (A) RT-qPCR analysis of transcript levels of several M1/1M2 markers in RAW264.7 cells treated with SjEVs. Representative results are shown, with means and standard errors from an experiment carried out in triplicate. * *P* ≤ 0.05 and ** *P* ≤ 0.01. (B). ELISA to determine the concentration of TNF-α, IL-10 and IL-13 released from RAW264.7 cells treated with SjEVs. The data shows representative results and illustrates the mean and standard errors from an experiment carried out in triplicate. * *P* ≤0.05 and ** *P* ≤ 0.01.
(TIF)

S12 Fig RT-qPCR analysis of the abundance of SjEV *miR-125b* and *bantam* in monocytes from the peripheral blood of mice administered clodronate liposomes or control liposomes. Representative results are shown, with means and standard errors from an experiment carried out in triplicate. * *P* ≤ 0.05 and ** *P* ≤ 0.01.
(TIF)

S1 Table List of the miRNAs identified in *S*. *japonicum* EVs. (XLSX)

S2 Table Differentially expressed genes in macrophages associated with SjEV treatment. (XLSX)

S3 Table KEGG analysis of differentially expressed genes in macrophages treated with SjEVs. (XLSX)

S4 Table List of the primers used for RT-qPCR analysis in the study. (XLSX)

S5 Table List of putative targets of SjEV *miR-125b* and *bantam* in down-regulated mRNAs. (XLSX)

S6 Table List of the primers used for miRNA target validation in the study. (XLSX)

S7 Table List of miRNA mimics/anti-sense miRNA used in the study. (XLSX)

S8 Table List of siRNAs for miRNA target silencing in the study. (XLSX)
