# Supplementary figures and images for: Schistosoma japonicum extracellular vesicle miRNA cargo regulates host macrophage functions facilitating parasitism
Source: PLoS Pathog. 2019 Jun 4;15(6):e1007817. doi: 10.1371/journal.ppat.1007817 (PMC6548406; doi:10.1371/journal.ppat.1007817)

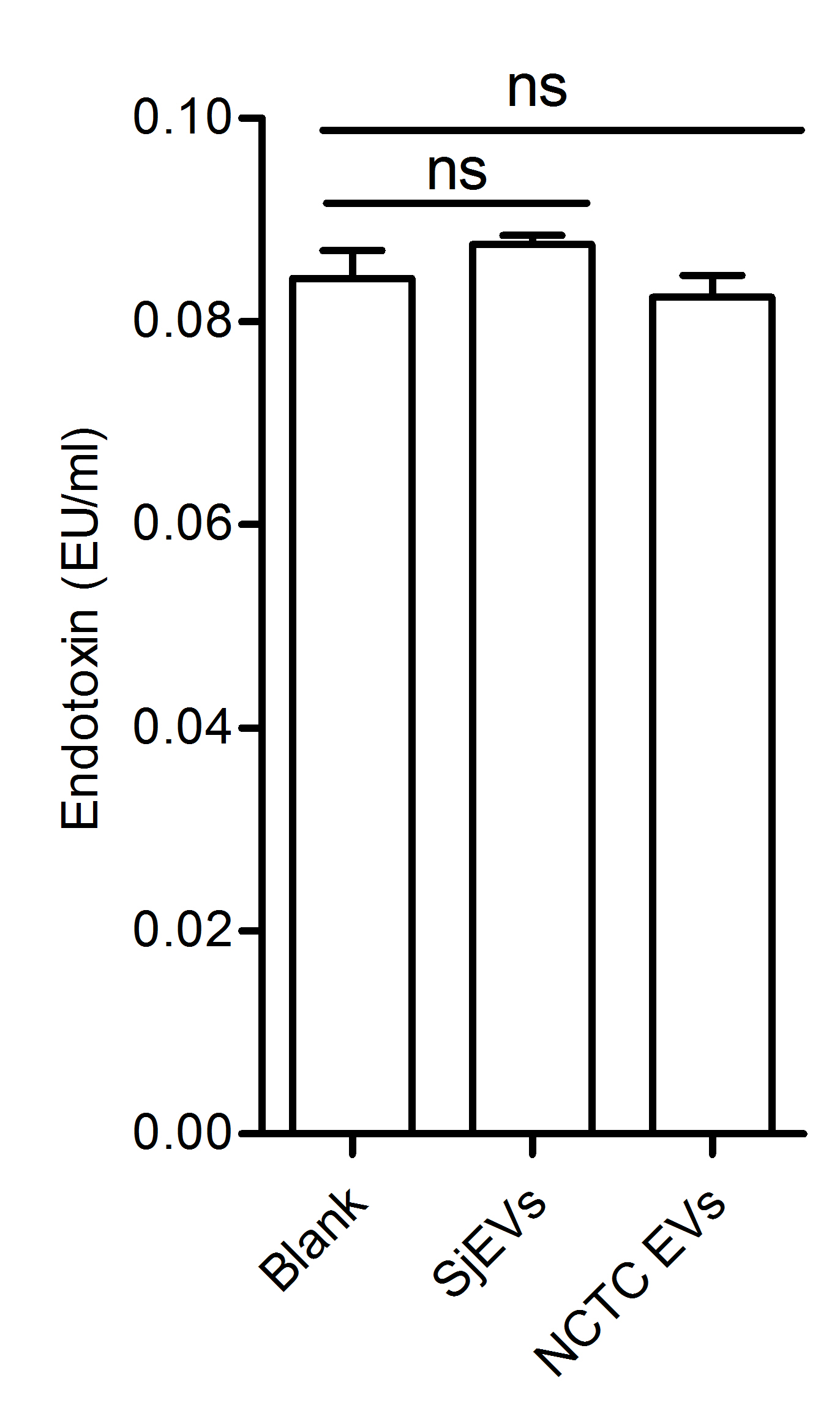

Supplement: S1 Fig — (TIF) [file ppat.1007817.s001.tif]

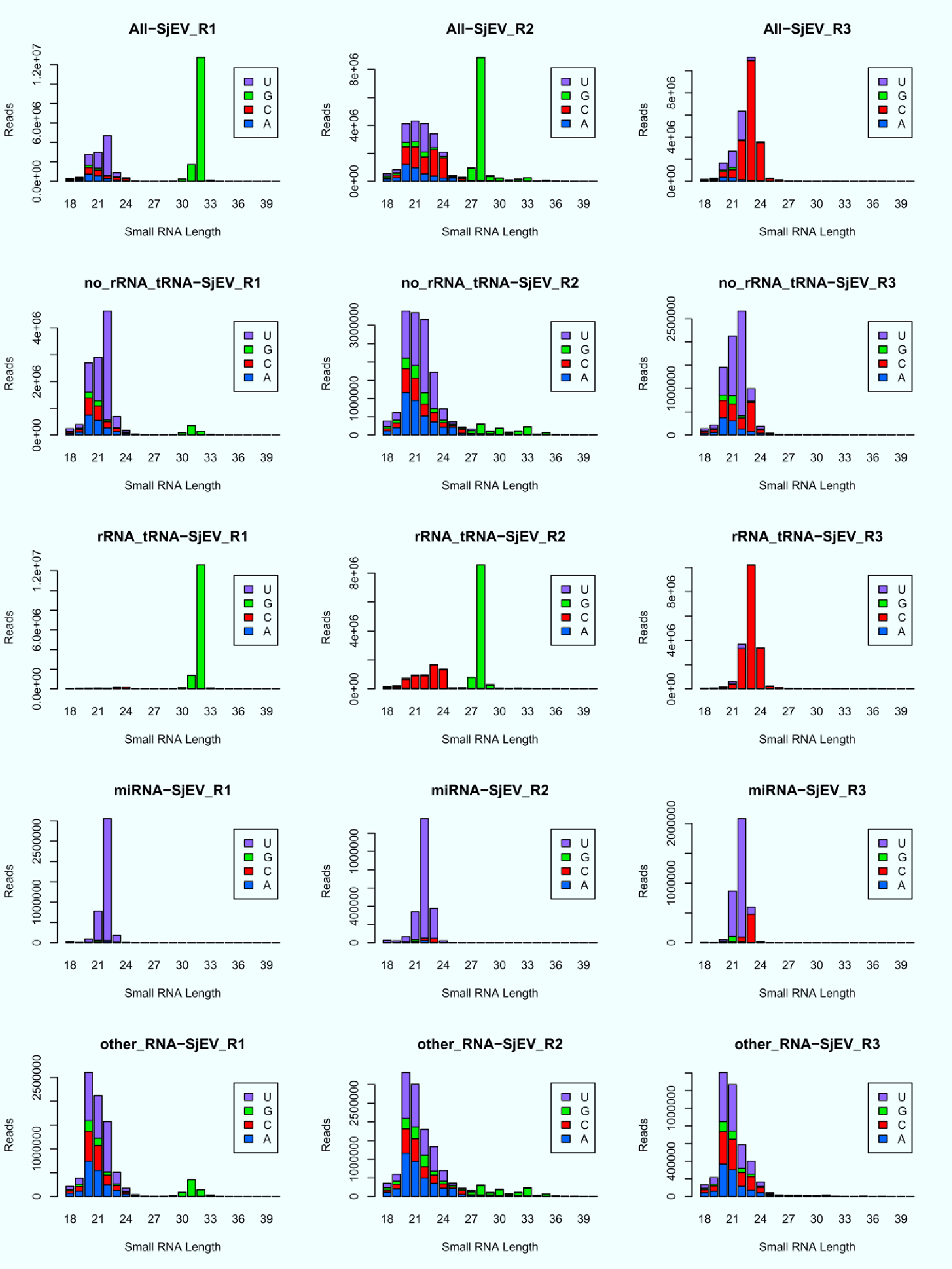

Supplement: S2 Fig — (TIF) [file ppat.1007817.s002.tif]

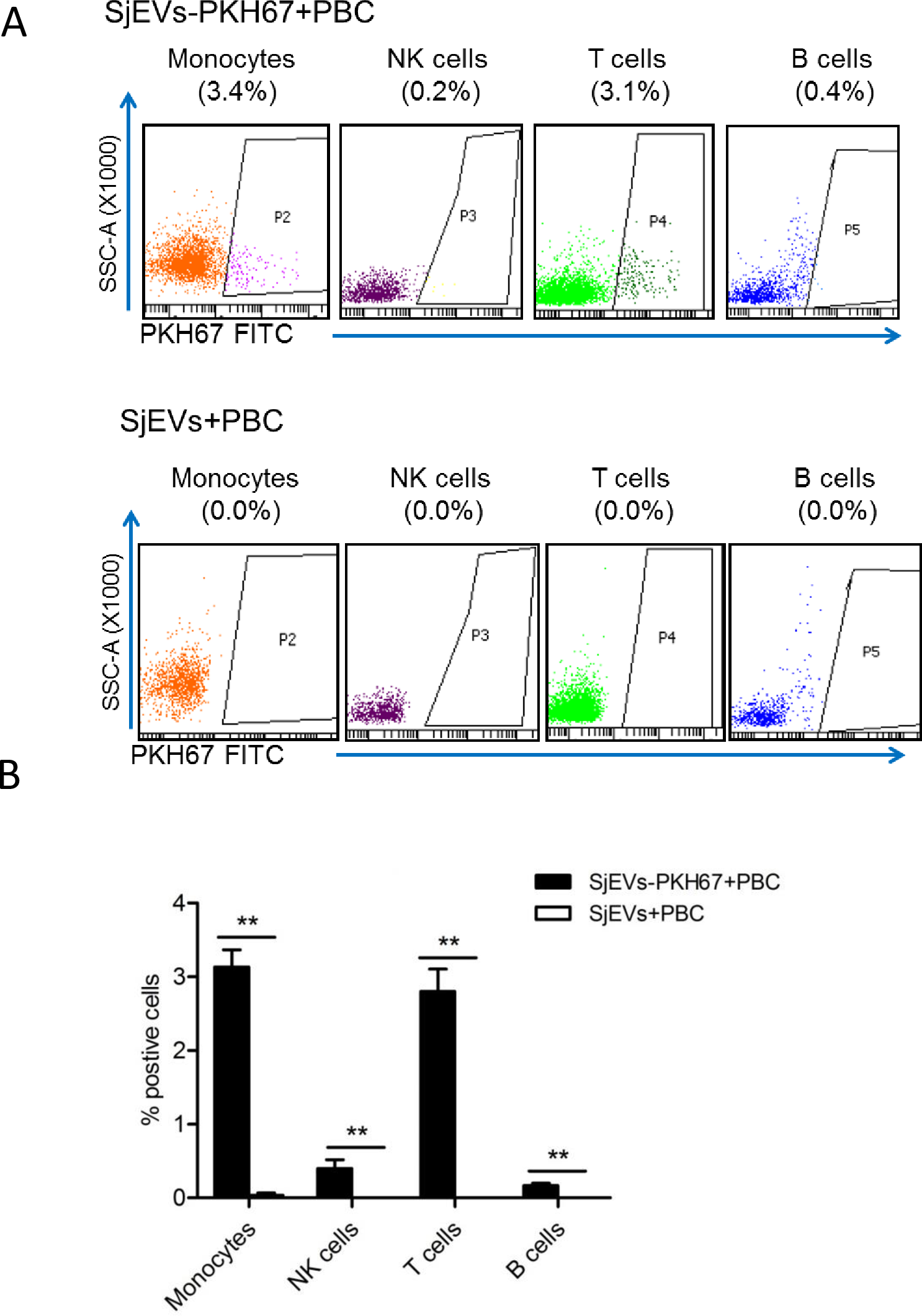

Supplement: S3 Fig — (A) Representative flow cytometry result analyzing SjEV uptake in different populations of peripheral blood immune cells in mice. The gating strategies were based on peripheral blood cell incubated unlabeled SjEVs. The results are representative of six independent experiments. (B) Quantitation of flow cytometry analysis of SjEV uptake in (A). Data illustrate representative results and show the mean and standard errors from six mice. * P ≤ 0.05 and ** P ≤ 0.01. (TIF) [file ppat.1007817.s003.tif]

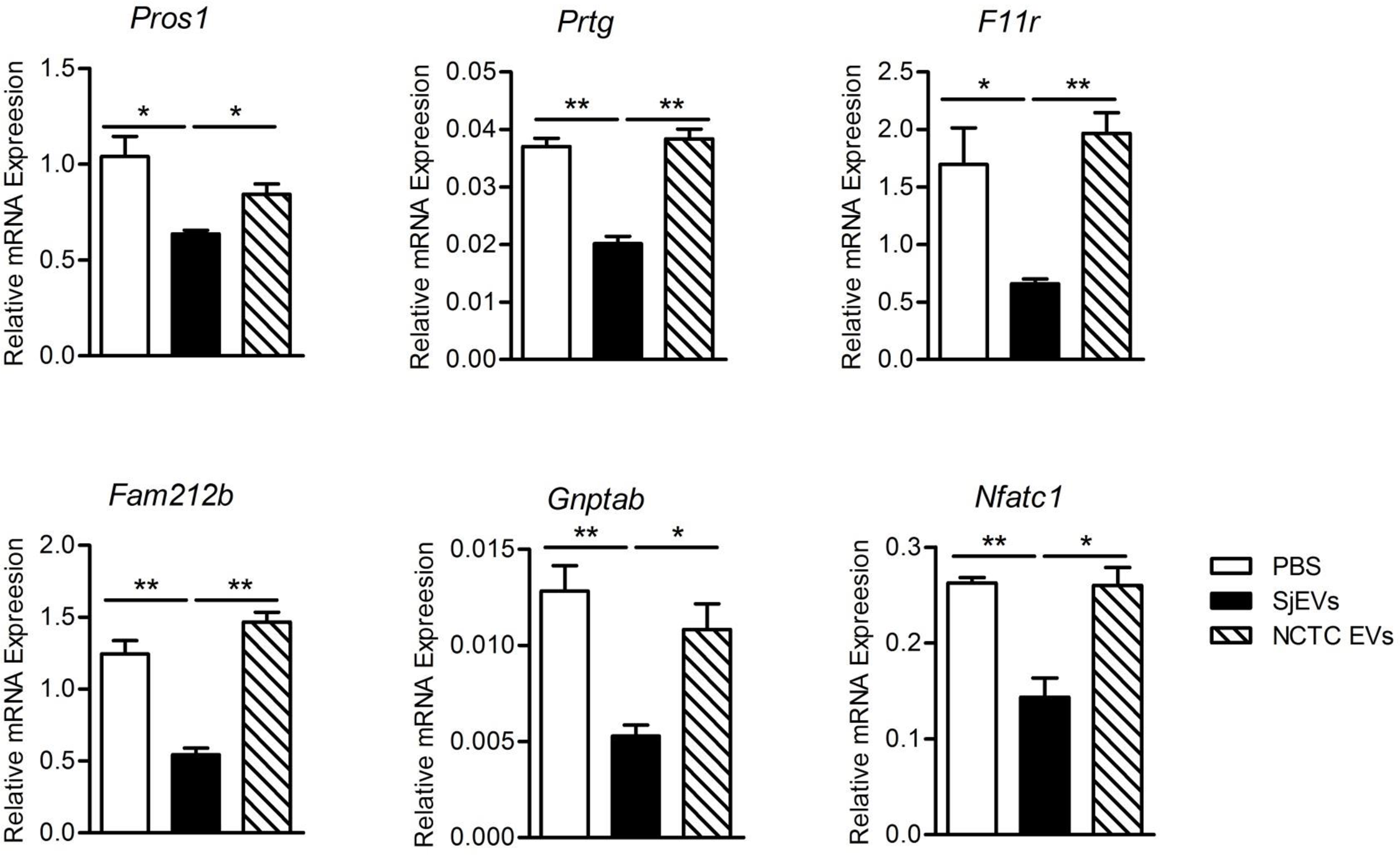

Supplement: S4 Fig — Data illustrate representative results and show the mean and standard errors from an experiment carried out in triplicate. * P ≤ 0.05 and ** P ≤ 0.01. (TIF) [file ppat.1007817.s004.tif]

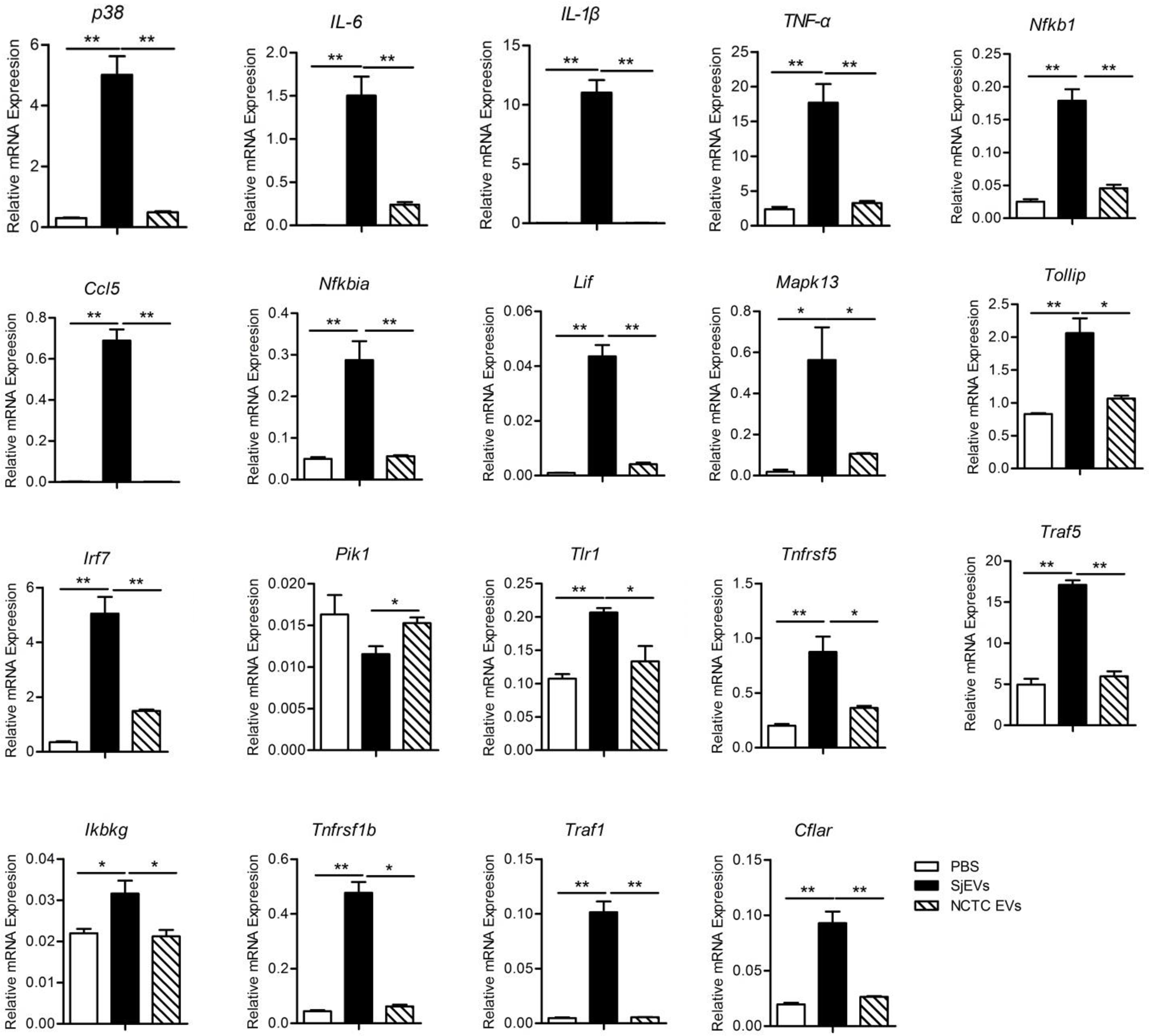

Supplement: S5 Fig — Data illustrate representative results and show the mean and standard errors from an experiment carried out in triplicate. * P ≤ 0.05 and ** P ≤ 0.01. (TIF) [file ppat.1007817.s005.tif]

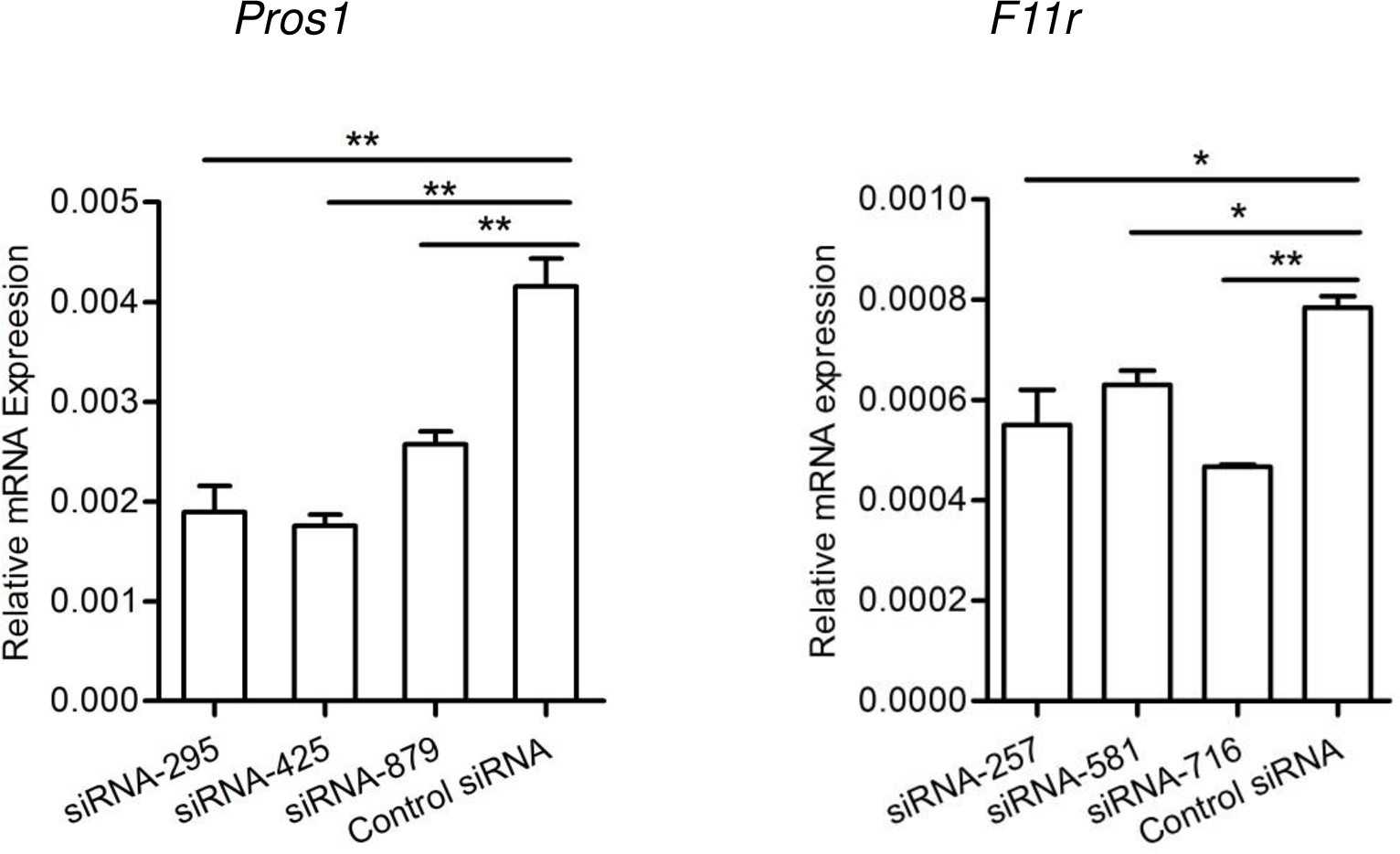

Supplement: S6 Fig — Each experiment shows representative results and illustrates the mean and standard errors from an experiment carried out in triplicate. * P ≤ 0.05 and ** P ≤ 0.01. (TIF) [file ppat.1007817.s006.tif]

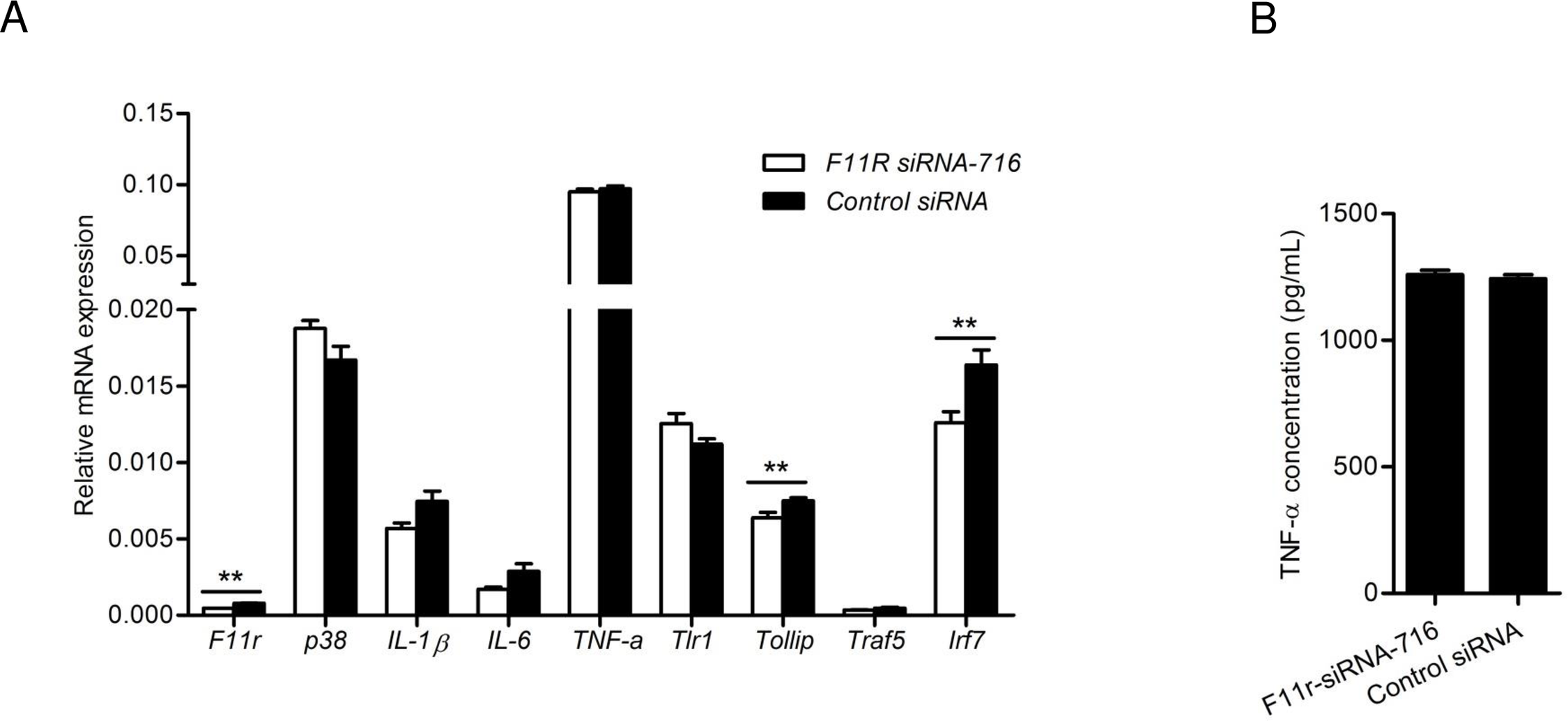

Supplement: S7 Fig — (A) RT-qPCR analysis of the expression of molecules involved in the TLR signaling pathway in RAW264.7 cells transfected with F11r siRNA-716. Each experiment shows representative results and illustrates the mean and standard errors from an experiment carried out in triplicate. * P ≤0.05. (B) ELISA for TNF-α concentration in the culture medium of RAW264.7 cells transfected with F11r siRNA-716. Each experiment shows representative results and illustrates the mean and standard errors from an experiment carried out in triplicate. (TIF) [file ppat.1007817.s007.tif]

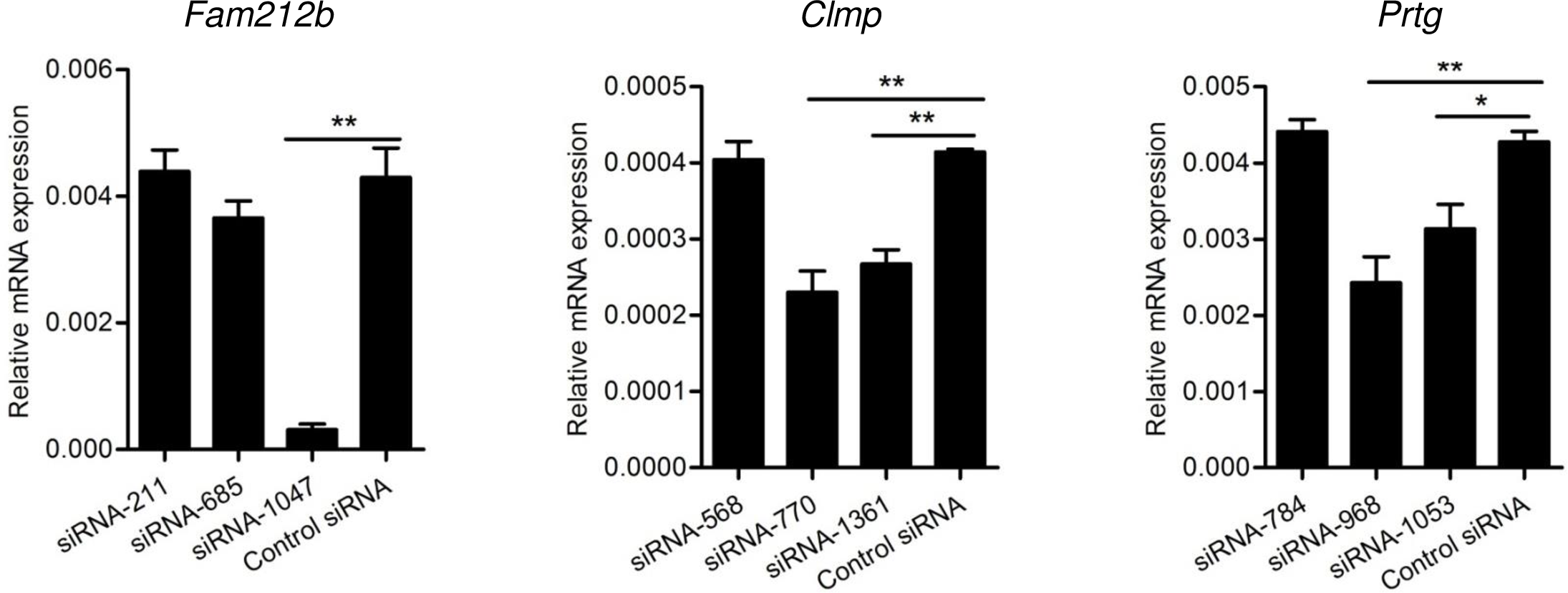

Supplement: S8 Fig — Each experiment shows representative results and illustrates the mean and standard errors from an experiment carried out in triplicate. * P ≤ 0.05 and ** P ≤ 0.01. (TIF) [file ppat.1007817.s008.tif]

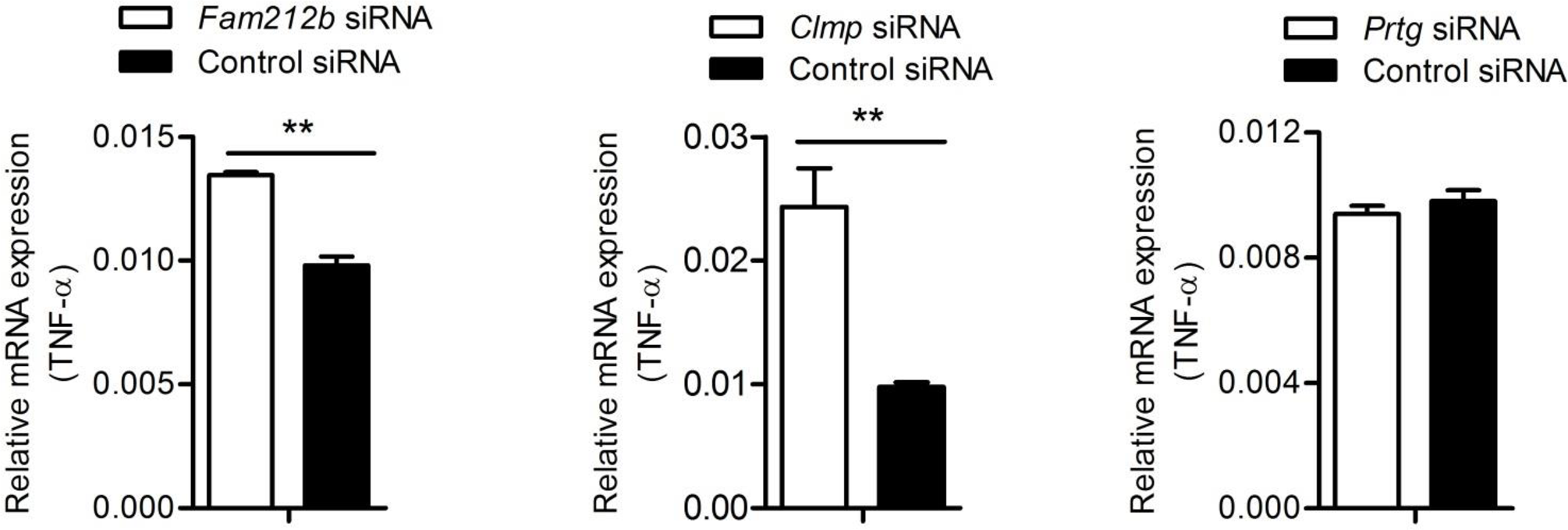

Supplement: S9 Fig — Data shows representative results and illustrates the mean and standard errors from an experiment carried out in triplicate. * P ≤ 0.05 and ** P ≤ 0.01. (TIF) [file ppat.1007817.s009.tif]

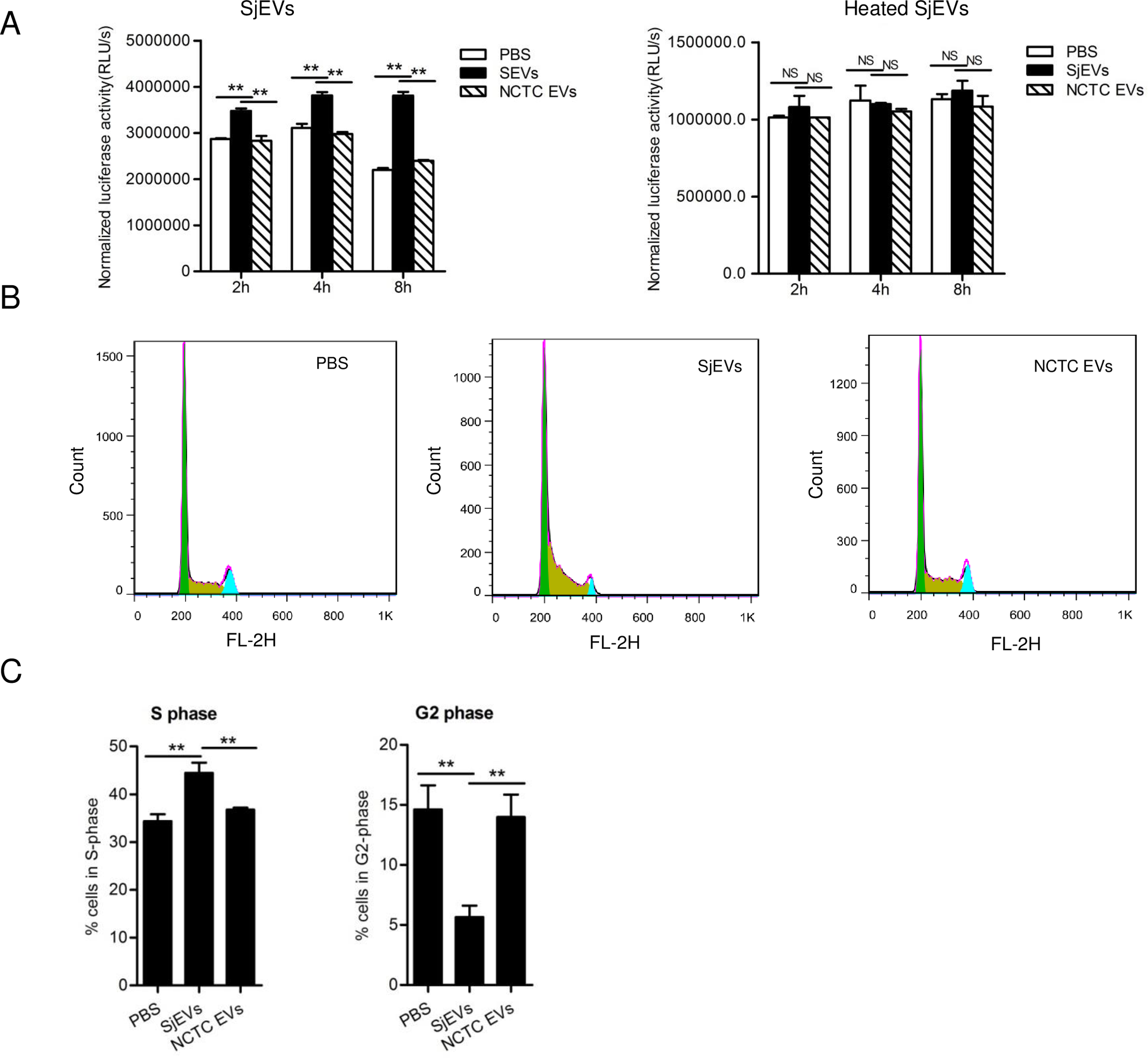

Supplement: S10 Fig — (A) SjEVs treatment of RAW264.7 cells increases their proliferation. At the indicated time of post treatment of SjEVs, RAW264.7 cells were collected and assayed using a cell Titer-Lumi luminescent cell viability kit. The luciferase activities indicated cell proliferation was increased as compared to that treated with heated inactivated SjEVs. Each experiment shows representative results and illustrates the mean and standard errors derived from triplicate experiments from an experiment carried out in triplicate. (B) and (C) SjEV treatment of RAW264.7 cells increases the population of cells in S phase. Each experiment shows representative results and illustrates the mean and standard errors from an experiment carried out in triplicate. (TIF) [file ppat.1007817.s010.tif]

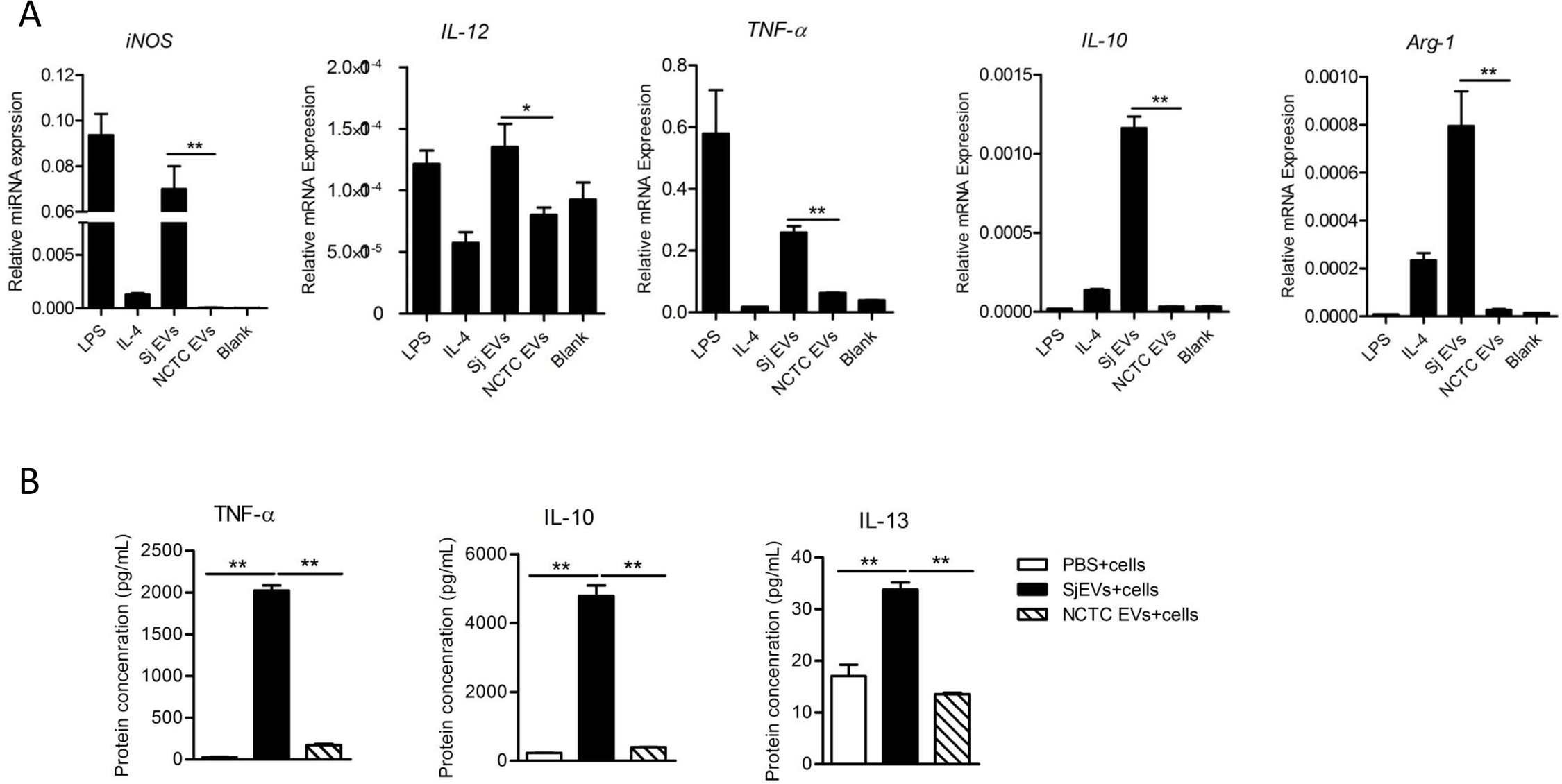

Supplement: S11 Fig — (A) RT-qPCR analysis of transcript levels of several M1/1M2 markers in RAW264.7 cells treated with SjEVs. Representative results are shown, with means and standard errors from an experiment carried out in triplicate. * P ≤ 0.05 and ** P ≤ 0.01. (B). ELISA to determine the concentration of TNF-α, IL-10 and IL-13 released from RAW264.7 cells treated with SjEVs. The data shows representative results and illustrates the mean and standard errors from an experiment carried out in triplicate. * P ≤0.05 and ** P ≤ 0.01. (TIF) [file ppat.1007817.s011.tif]

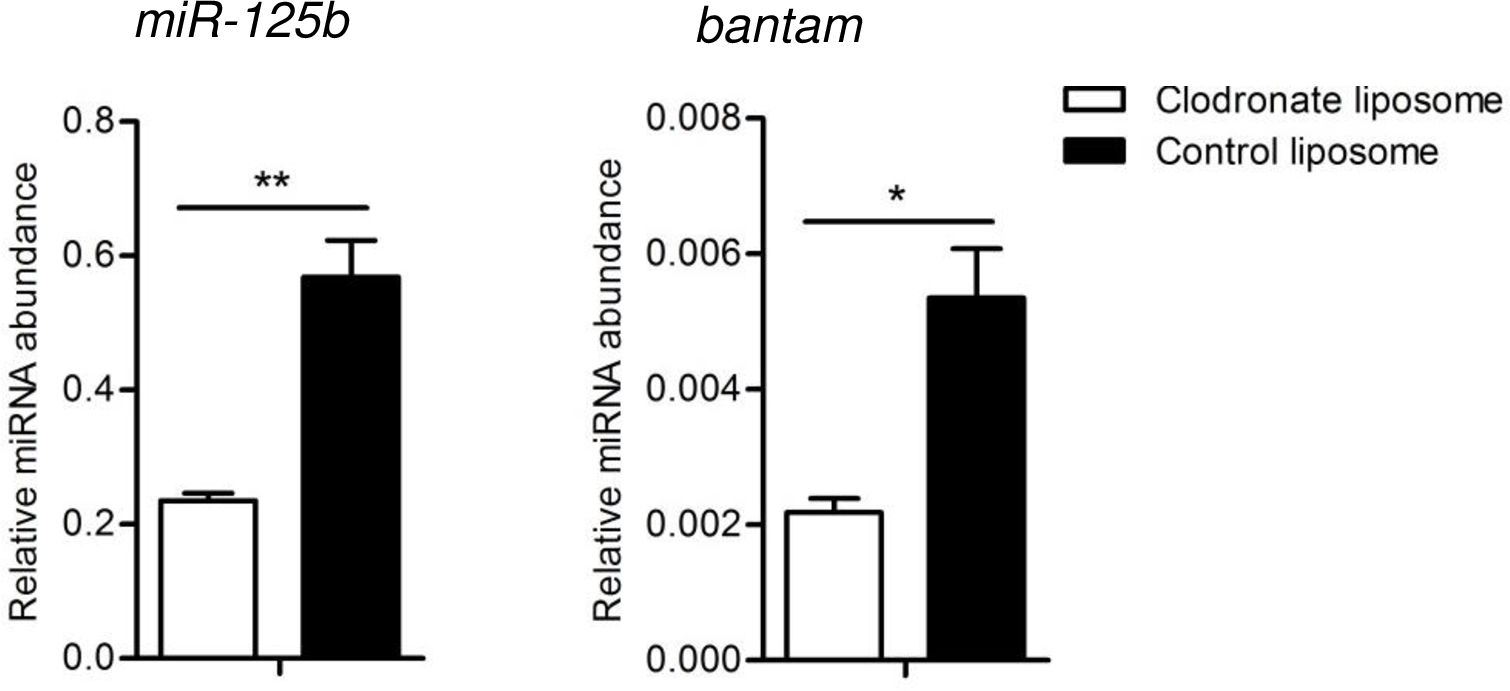

Supplement: S12 Fig — Representative results are shown, with means and standard errors from an experiment carried out in triplicate. * P ≤ 0.05 and ** P ≤ 0.01. (TIF) [file ppat.1007817.s012.tif]
